# Permissive microbiome characterizes human subjects with a neurovascular disease cavernous angioma

**DOI:** 10.1038/s41467-020-16436-w

**Published:** 2020-05-27

**Authors:** Sean P. Polster, Anukriti Sharma, Ceylan Tanes, Alan T. Tang, Patricia Mericko, Ying Cao, Julián Carrión-Penagos, Romuald Girard, Janne Koskimäki, Dongdong Zhang, Agnieszka Stadnik, Sharbel G. Romanos, Seán B. Lyne, Robert Shenkar, Kimberly Yan, Cornelia Lee, Amy Akers, Leslie Morrison, Myranda Robinson, Atif Zafar, Kyle Bittinger, Helen Kim, Jack A. Gilbert, Mark L. Kahn, Le Shen, Issam A. Awad

**Affiliations:** 1 Section of Neurosurgery, Department of Surgery, The University of Chicago, 5841S. Maryland Avenue, Chicago, IL 60637 USA; 2 Department of Surgery, The University of Chicago, 5841S. Maryland Avenue, Chicago, IL 60637 USA; 3 Department of Pediatrics, The University of California San Diego and Scripps Institution of Oceanography, 9500 Gilman Drive, La Jolla, CA 92093 USA; 4 Division of Gastroenterology, Hepatology, and Nutrition, Children’s Hospital of Philadelphia, 2716 South Street, Philadelphia, PA 19146 USA; 5 Department of Medicine and Cardiovascular Institute, University of Pennsylvania, 3400 Civic Center Boulevard, Philadelphia, PA 19104 USA; 6 Department of Anesthesia and Perioperative Care, Center for Cerebrovascular Research, 513 Parnassus Avenue, San Francisco, CA 94143 USA; 7 Angioma Alliance, 520W 21st Street, Norfolk, VA 23517 USA; 8 Department of Neurology, University of New Mexico, 1 University of New Mexico, Albuquerque, NM 87131 USA

**Keywords:** Microbiome, Biomarkers, Cerebrovascular disorders

## Abstract

Cavernous angiomas (CA) are common vascular anomalies causing brain hemorrhage. Based on mouse studies, roles of gram-negative bacteria and altered intestinal homeostasis have been implicated in CA pathogenesis, and pilot study had suggested potential microbiome differences between non-CA and CA individuals based on 16S rRNA gene sequencing. We here assess microbiome differences in a larger cohort of human subjects with and without CA, and among subjects with different clinical features, and conduct more definitive microbial analyses using metagenomic shotgun sequencing. Relative abundance of distinct bacterial species in CA patients is shown, consistent with postulated permissive microbiome driving CA lesion genesis via lipopolysaccharide signaling, in humans as in mice. Other microbiome differences are related to CA clinical behavior. Weighted combinations of microbiome signatures and plasma inflammatory biomarkers enhance associations with disease severity and hemorrhage. This is the first demonstration of a sensitive and specific diagnostic microbiome in a human neurovascular disease.

## Abstract (web summary)

Gut microbiome has been linked to cavernous angioma (CA), a common vascular disease, but the role in humans remains unclear. Here, the authors combine 16S rRNA sequencing and shotgun metagenomics to profile the microbiome in a large cohort of human subjects with and without CA, and among subjects with different CA clinical features.

## Introduction

Cavernous angiomas (CAs) are characterized by dysmorphic dilated vascular capillaries, or caverns, lined by endothelium^1–3^. About 30–40% of CA patients have autosomal dominant inherited germline mutations in one of three known CA genes (*CCM1*/*KRIT1*, *CCM2*/*Malcavernin*, and *CCM3*/*PDCD10*), and develop multifocal lesions throughout the brain and spinal cord. Patients with the sporadic form of the disease manifest solitary CAs in the absence of germline mutations^4–9^. Lesional endothelial cells in sporadic CAs harbor somatic mutations of the same genes implicated in the familial disease^10^. *CCM* gene-encoded proteins assemble to form a trimeric complex^11^. The function of this complex can be disrupted by mutation of any of the three genes^11^, which could explain similar brain lesions in patients with mutations in different *CCM* genes, and in sporadic and familial cases.

The severity and natural course are highly variable among CA patients even with the same genetic mutation^1,2,12–14^. Such variation includes the number of lesions in familial cases, age at first symptomatic manifestation, and propensity to have symptomatic bleeds. It has been increasingly recognized that CA disease course can be affected by inflammatory processes. Immune cells accumulate within CA lesions^15^, B cell depletion blunts lesion development and bleeding in mouse models^16^, and plasma inflammatory cytokines may be used to differentiate CA patients with distinctive disease characteristics^17–19^. Furthermore, a genome-wide association study of CA patients with the same common Hispanic mutation of *KRIT1* showed that CA lesion number is associated with pro-inflammatory genotypes^13^, including polymorphisms in the lipopolysaccharide (LPS) receptors TLR4 and CD14^20^.

A gut–brain axis has been postulated in CA disease, based on studies in murine models. Inhibiting TLR4 pathway pharmacologically or through endothelial-specific TLR4 deletion significantly reduces, while LPS injection increases, brain lesion burden in *Ccm*-deleted mice, by affecting CCM complex-controlled TLR4-MEKK3-KLF2/4 signaling^20^. Germ-free *Ccm*-deleted mice, those treated with antibiotics, and mice with resistant microbiota all have significantly lower lesion volume than mice with susceptible microbiota^20^. The susceptible mouse microbiome is associated with increased LPS-producing Gram-negative bacteria Bacteroidetes family member S24-7, and this has been linked to LPS-induced TLR4 signaling^20^. Other work identified enhanced epithelial permeability in association with *Ccm1* depletion^21^, and grossly impaired gut barrier in association with the more severe *Ccm3* deletion^22^. Pilot study also suggested greater prevalence of Gram-negative bacteria in the gut microbiome of patients with CAs^22^.

To date, sensitivity and specificity of a potential diagnostic microbiome have not been shown in human subjects with CA, and it is not known what bacterial species might contribute to this disease. It remains unclear whether a diagnostic microbiome differentiates familial/multifocal cases as well as the more common cases with sporadic/solitary CAs (without germline mutations in *CCM* genes). Furthermore, variations in microbiome have never been correlated with CA clinical manifestations, nor analyzed in relation to plasma biomarkers implicated in disease severity.

Our study demonstrates that CA patients have distinctive microbiome compared to healthy individuals. Analyses at the biosynthesis and gene level indicate that LPS synthesis-related genes are more abundant in CA patients, consistent with a role of gut-generated LPS driving CA disease. This study further shows that CA patients with distinct disease characteristics have different microbiota, and that the combination of plasma biomarker and stool microbiome composition enhances this differentiation.

## Results

### Microbiome distinguishes human subjects with and without CA

**Fig. 1:**
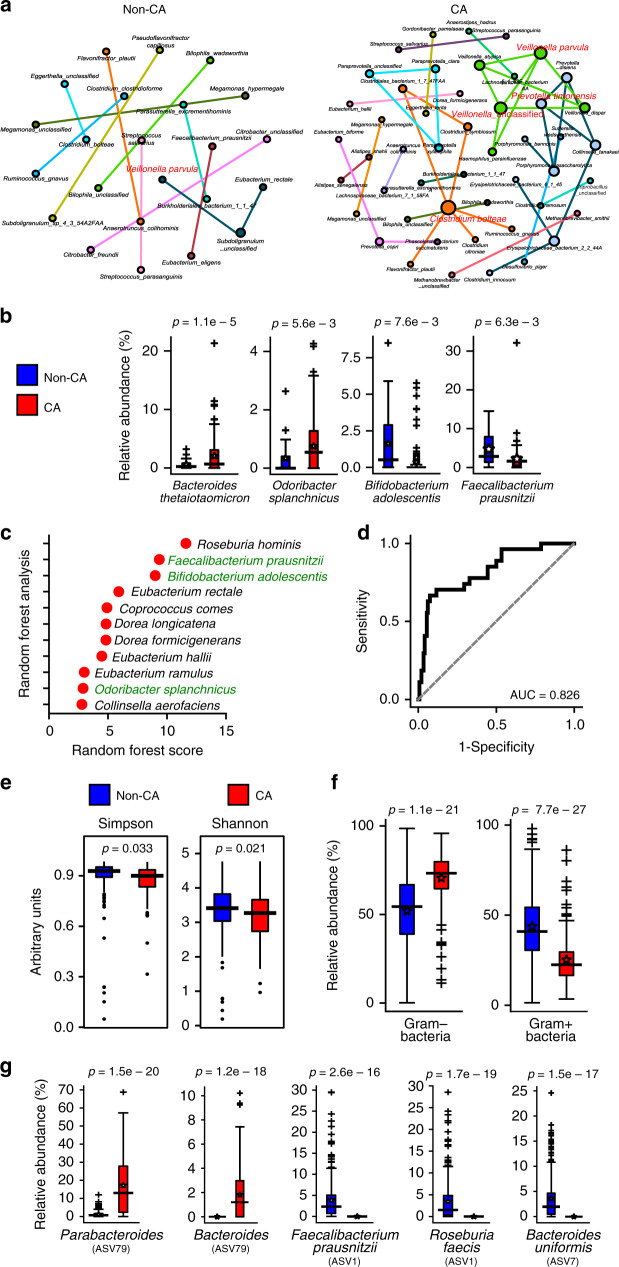
Fecal microbiotas are different in CA and non-CA cohorts. **a** Organization of microbiome species in non-CA and CA cohorts. Co-occurrence network analyses were performed at the species level, as determined by metagenomic shotgun sequencing data analysis (*n* = 27 non-CA, *n* = 122 CA, keystone species are labeled in red). **b** Multi-variate differential abundance analyses of metagenomic shotgun data at species level. Species with significantly different abundance (ANCOM analysis, followed by two-sided Mann–Whitney *U* test with Benjamini–Hochberg false discovery rate (FDR) correction for multiple testing, p_FDR_ < 0.01) and medium relative abundance ≥0.25% in either group are presented as box-whisker plots (blue boxes: non-CA cohort; red boxes: CA cohort). **c** Identification of key species (medium relative abundance ≥0.1% in either group) by random forest analysis. Species identified in both multi-variate and random forest analyses are shown in green. **d** ROC curve was identified based on best-weighted combination of common bacterial species identified by multi-variate and random forest analyses (AUC = 0.826, specificity = 0.667, sensitivity = 0.925). **e** α-Diversity analyses of fecal samples by Shannon and Simpson indices based on 16 S rRNA gene amplicon sequencing data, presented as box-whisker plots (*n* = 250 non-CA, *n* = 115 CA, Kruskal–Wallis one-way analysis of variance test; blue boxes: non-CA cohort; red boxes: CA cohort). **f** Relative abundance of Gram-negative and Gram-positive bacteria in non-CA and CA cohorts (*n* = 250 non-CA, *n* = 115 CA, ANCOM analysis, followed by two-sided Mann–Whitney *U* test, blue boxes: non-CA cohort, red boxes: CA cohort). **g** Multi-variate differential abundance taxonomic analyses of 16S rRNA gene amplicon sequencing data. ESVs with significantly different relative abundances (p_FDR_ ≤ 0.01) and medium relative abundance of ≥1% in either group are presented as box-whisker plots (*n* = 250 non-CA, *n* = 115 CA, ANCOM analysis, followed by two-sided Mann–Whitney *U* test with Benjamini–Hochberg FDR correction; blue boxes: non-CA cohort; red boxes: CA cohort). In box plots, bounds of boxes show interquartile range (IQR), top and bottom whiskers demonstrate maximum and minimum, lines in the middle of the box indicate median, and stars show mean of the data. + signs indicate outliers.

A pilot 16S ribosomal RNA (rRNA) amplicon analysis based on operational taxonomic unit calling had suggested microbiome differences between CA and non-CA individuals^22^. To compare the microbiome differences in more detail and with better confidence, we recruited a larger cohort with greater representation of CAs with various genotypes. We performed metagenomic shotgun sequencing analysis and taxonomic identification of genome clusters (Fig. 1a–d). For further confirmation, we also compared the microbiome of CA patients using the 16S rRNA amplicon sequencing data and the publicly available American Gut Project data of age- and gender-matched non-CA individuals with exact sequence variation (ESV) taxonomical calling (Fig. 1e–g)^23,24^. Co-occurrence network analyses of metagenomic shotgun sequencing of the proportional differences of each taxon demonstrated significant differences in the connectivity of the network clusters between CA and non-CA individuals, which were supported by different keystone species between CA and non-CA networks (Fig. 1a). Such population differences were supported by the differences in both α- and β-diversity (*p* ≤ 0.05) between CA and non-CA individuals based on 16S rRNA amplicon analysis, suggesting substantial shifts in the richness and proportions of community members (Fig. 1e, Supplementary Fig. 1a, b). The trend for genera *Bacteroides* and *Faecalibacterium* were similar irrespective of the analytical method and using different control populations, indicating they are robust biomarkers (Fig. 1b, g, Supplementary Fig. 1c–e).

Three common species were identified by combining multi-variate and machine learning-based random forest analyses of metagenomic shotgun data. The Gram-negative *Odoribacter splanchnicus* was significantly increased (false discovery rate corrected *p* value (p_FDR_) ≤ 0.05), and the Gram-positive *Faecalibacterium prausnitzii* and *Bifidobacterium adolescentis* were significantly decreased (p_FDR_ ≤ 0.05) in CA patients (Fig. 1b, c). Based on receiver operating characteristic (ROC) curves, the optimal weighted combination of these three species provided good sensitivity (92%) and specificity (67%) in association with CA diagnosis (Fig. 1d). Notably, CA patients’ microbiota were significantly enriched in the proportion of Gram-negative bacteria (*p* ≤ 0.05, Fig. 1f), consistent with our previous animal model study^20^. Similar bacterial species respectively distinguished sporadic/solitary and familial/multifocal CA cases from non-CA subjects (Supplementary Fig. 2). There were no batch effects among cases recruited at various sites or during different phases of patient recruitment (Supplementary Fig. 7 and Supplementary Discussion). Among familial/multifocal CA patients, those with different *CCM* gene mutations had limited microbiome differences (Supplementary Fig. 3 and Supplementary Discussion). Analysis by gender did not suggest significant confounders (Supplementary Fig. 5 and Supplementary Discussion).

### CA microbiome is enriched with LPS synthesis-related genes

**Fig. 2:**
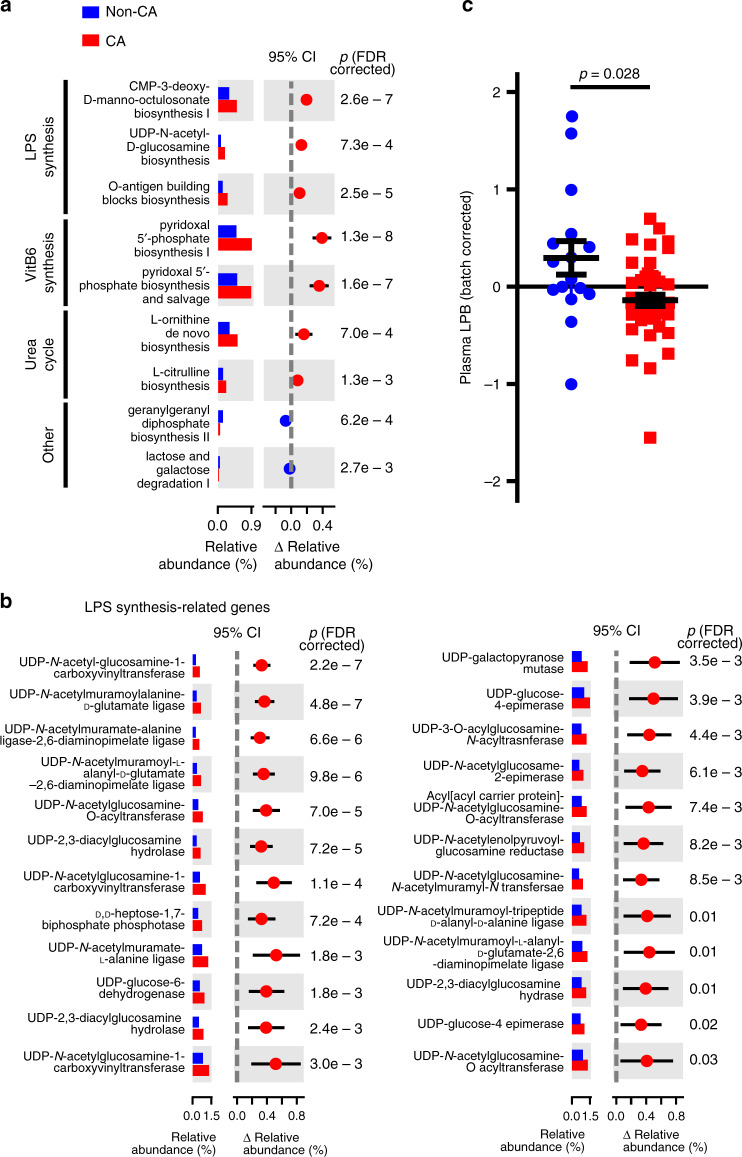
Fecal LPS synthesis pathway is upregulated in CA patients. **a** Metagenomic shotgun data were used to compare relative gene abundance of bacterial metabolic pathways. Relative abundances of significantly different metabolic pathways are shown (*n* = 27 non-CA, *n* = 121 CA, two-sided Mann–Whitney *U* test with Benjamini–Hochberg FDR correction). **b** Relative abundance of significantly different LPS synthesis pathway-related genes between non-CA and CA individuals (two-sided Mann–Whitney *U* test with Benjamini–Hochberg FDR correction). **c** Batch-corrected LPB content in peripheral plasma of non-CA and CA individuals (*n* = 16 for non-CA, *n* = 47 for CA individuals, two-tailed unpaired *t* test with Welch’s correction). Average data are presented as mean ± s.e.m. Blue: non-CA individuals, red: CA patients.

Metagenomic shotgun sequencing data were also used to reconstruct and compare metabolic pathways in both CA and non-CA samples. Untargeted analysis identified LPS biosynthesis-related pathways were significantly enriched in CA samples (p_FDR_ ≤ 0.05) (Fig. 2a), consistent with a role for Gram-negative bacteria produced LPS to promote CA-like lesion formation demonstrated in mouse studies^20^. Furthermore, the relative abundance of many LPS biosynthesis-related genes by targeted analysis were significantly more abundant in the CA microbiome (p_FDR_ ≤ 0.05, Fig. 2b). Genes associated with vitamin B6 biosynthesis and the urea cycle were also significantly enriched (p_FDR_ ≤ 0.05) in the CA-associated microbiome (p_FDR_ ≤ 0.05, Fig. 2a), possibly reflecting additional mechanisms of action. Furthermore, the levels of LPS-binding protein (LPB) in plasma, a protein frequently downregulated when blood LPS content is increased^25–27^, were lower in CA patients relative to non-CA patients (Fig. 2c). Taken together, these analyses show that the CA-permissive microbiome is enriched with LPS-producing Gram-negative bacteria.

### Microbiome differences in CA subpopulations

**Fig. 3:**
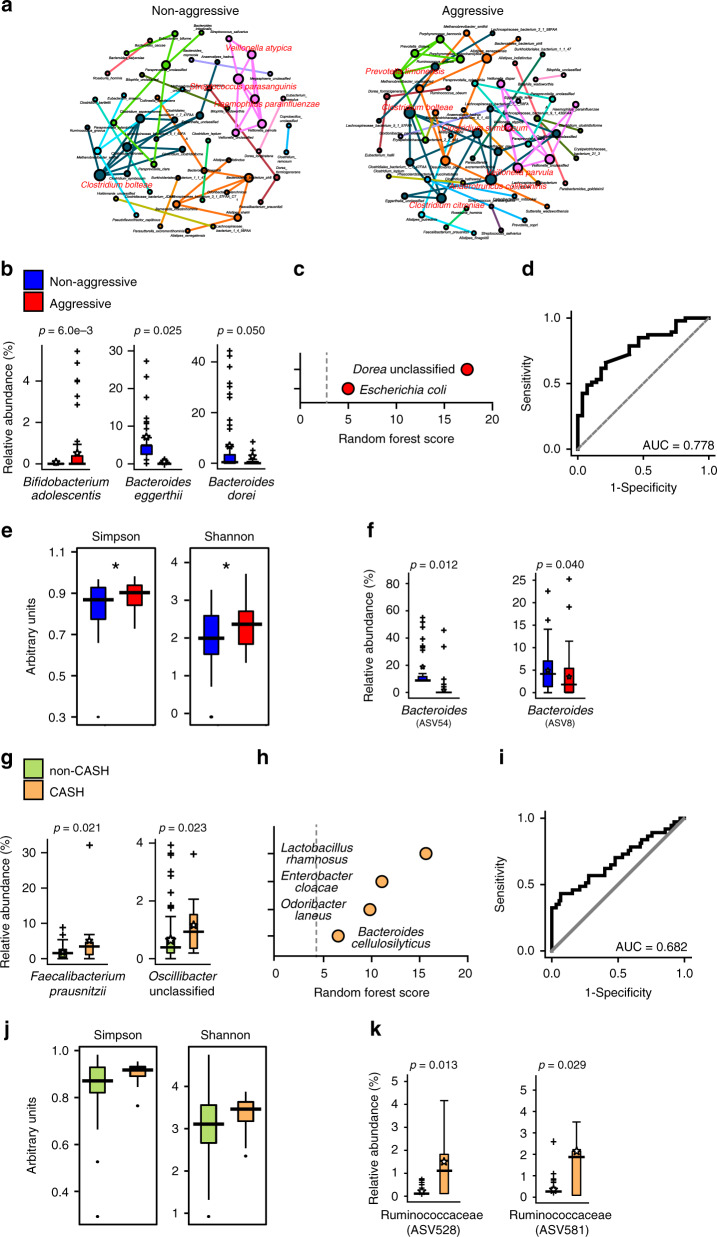
CA patient subpopulations can have different microbiota. **a** Organization of microbiome species in non-aggressive and aggressive patients. Co-occurrence network analyses were performed at species level, as determined by metagenomic shotgun data analysis (*n* = 45 non-aggressive patients, *n* = 62 aggressive patients, keystone species are labeled in red). **b** Multi-variate differential abundance analysis of metagenomic shotgun data between non-aggressive and aggressive patients at species level. Species with significantly different abundance are presented as box-whisker plots (ANCOM analysis followed by two-sided Mann–Whitney *U* test with Benjamini–Hochberg FDR correction, blue boxes: non-aggressive patients, red boxes: aggressive patients). **c** Identification of key species by random forest analysis. **d** ROC curve was identified based on best-weighted combination of all bacterial species identified by multi-variate and random forest analyses (AUC = 0.778, specificity = 0.786, sensitivity = 0.660). **e** α-Diversity analyses of fecal samples of CA patients with non-aggressive and aggressive disease by Shannon and Simpson indices based on 16S rRNA gene amplicon sequencing data (*n* = 43 non-aggressive patients, *n* = 58 aggressive patients, Kruskal–Wallis one-way analysis of variance test, blue boxes: non-aggressive patients, red boxes: aggressive patients). **f** Multi-variate differential abundance taxonomic analyses between non-aggressive and aggressive CA patients based on 16S rRNA gene amplicon sequencing results. ESVs with significantly different relative abundances are presented as box-whisker plots (ANCOM analysis followed by two-sided Mann–Whitney *U* test with Benjamini–Hochberg FDR correction, blue boxes: non-aggressive patients, red boxes: aggressive patients). **g** Multi-variate differential abundance analysis of metagenomic shotgun data between non-CASH and CASH patients at the species level. Species with significantly different abundance are presented as box-whisker plots (*n* = 100 non-CASH patients, *n* = 13 CASH patients, ANCOM analysis followed by two-sided Mann–Whitney U test with Benjamini–Hochberg FDR correction, green boxes: non-CASH patients, orange boxes: CASH patients). **h** Identification of key species by random forest analysis. **i** ROC curve was identified based on best-weighted combination of all bacterial species identified by multi-variate and random forest analyses (AUC = 0.682, specificity = 0.933, sensitivity = 0.432). **j** α-Diversity analyses of fecal samples of CA patients with non-CASH and CASH disease by Shannon and Simpson indices based on 16S rRNA gene amplicon sequencing data, presented as box-whisker plots (*n* = 93 non-CASH patients, *n* = 13 CASH patients, Kruskal–Wallis one-way analysis of variance test, green boxes: non-CASH patients, orange boxes: CASH patients). **k** Multi-variate differential abundance taxonomic analyses between non-CASH and CASH patients based on 16S rRNA gene amplicon sequencing results. ESVs with significantly different relative abundances are presented as box-whisker plots (ANCOM analysis followed by two-sided Mann–Whitney *U* test with Benjamini–Hochberg FDR correction, green boxes: non-CASH patients, orange boxes: CASH patients). In box plots, bounds of boxes show IQR, top and bottom whiskers demonstrate maximum and minimum, lines in the middle of the box indicate median, and stars show mean of the data. + signs indicate outliers.

We have previously designed a classification system to categorize patients as having aggressive or non-aggressive disease over their lifetime, based on magnetic resonance imaging (MRI) counts of large lesions, age of symptom onset, and the number of symptomatic hemorrhages^17^. Metagenomic shotgun co-occurrence network analyses suggested that aggressive and non-aggressive CA patients have different network connectivity and unique keystone species (Fig. 3a). Multi-variate differential abundance analyses and random forest classifier identified five significantly contributive taxa, *Bifidobacterium adolescentis*, *Bacteroides eggerthii, Bacteroides dorei*, *Dorea*, and *Escherichia coli* (Fig. 3b, c, Supplementary Fig. 4c). Such microbiome differences moderately distinguish aggressive vs. non-aggressive CA patients (Fig. 3d). Consistent with metagenomic shotgun sequencing analysis, 16S rRNA amplicon sequencing data suggest that α- and β-diversity significantly differ between aggressive and non-aggressive CA patients (Fig. 3e, Supplementary Fig. 4a, b), with a significant decrease (p_FDR_ ≤ 0.05) in the proportion of two ESVs belonging to genus *Bacteroides* in aggressive disease (Fig. 3f).

CA patients with radiographically and clinically defined symptomatic hemorrhage (CASH) in the prior year are at higher risk of future bleeding^2,14^. These patients are likely to undergo invasive and risky surgical treatments. We tested if CASH patients have a unique microbiome signature. Co-occurrence network analysis was able to construct a significant network (*R*^2^ > 0.6, *p* < 0.05) for non-CASH patients, but not CASH patients. Multi-variate differential abundance analyses and random forest analyses identified six taxa including *Faecalibacterium prausnitzii*, *Oscillobacter*, *Lactobacillus rhamnosus*, *Enterobacter cloacae*, *Odoribacter laneus*, and *Bacteroides cellulosilyticus* (Fig. 3g, h, Supplementary Fig. 4i), whose best-weighted combination generated an ROC curve that moderately distinguishes CASH and non-CASH patients (Fig. 3i). Microbiome associations of disease severity and hemorrhage appear different and more complex than LPS mechanisms reflecting CA diagnosis (i.e., lesion development), and these require further investigations.

### Combined contributions of microbiome and plasma biomarkers

**Fig. 4:**
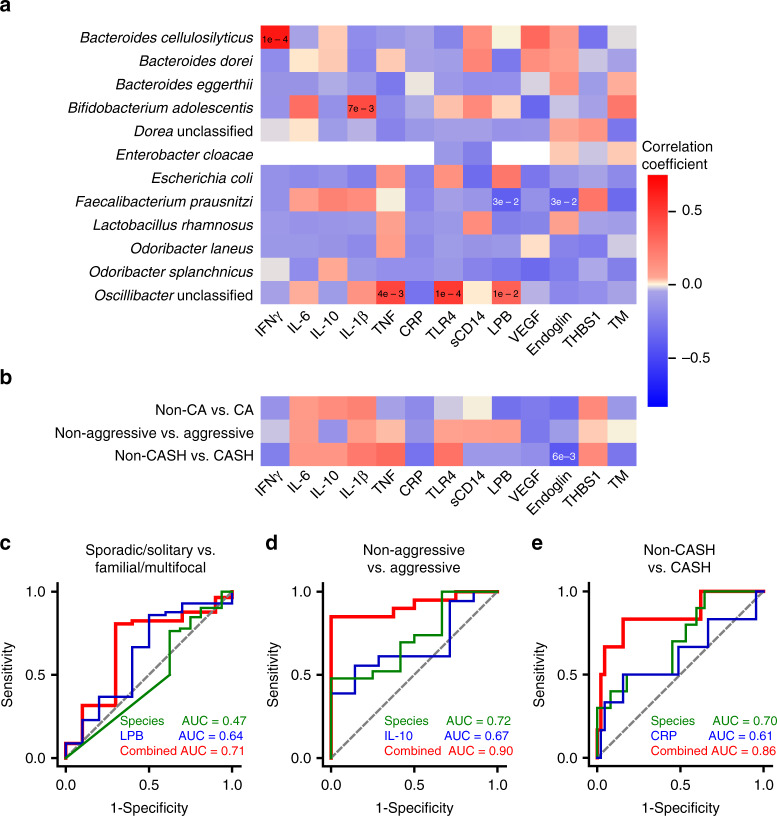
Plasma and fecal microbiome as CA biomarkers. **a** Correlation between individual bacterial species and circulating factors. Correlation between species identified by multi-variate differential abundance and random forest analyses (Figs. 1 and 3, Supplementary Fig. 2) and individual plasma biomarkers. Degree of correlation is color coded (also see Supplementary Table 1, Pearson’s correlation testing; red: positive correlation; blue: negative correlation). **b** Correlation between combination of species identified based on clinical questions and circulating factors. Correlation between combination of bacterial species identified (in Figs. 1 and 3, Supplementary Fig. 2) and individual plasma biomarkers. Degree of correlation is color coded (also see Supplementary Table 1, Pearson’s correlation testing, red: positive correlation, blue: negative correlation). **c**–**e** Comparison of best ROC curve determined by combination of bacterial species identified in Fig. 3 and Supplementary Fig. 2, individual circulating factors, and bacterial species and individual circulating factors. **c** Distinction between sporadic/solitary and familial/multifocal CA patients (green: bacterial species identified in Supplementary Fig. 2 (*B. dorei*), blue: LPB, red: combined bacterial species and LPB). **d** Distinction between non-aggressive and aggressive CA patients (green: bacterial species identified in Fig. 3b, c, blue: IL-10, red: combined bacterial species and IL-10). **e** Distinction between non-CASH and CASH patients (CASH patient, green: bacterial species identified in Fig. 3g, h, blue: CRP, red: combined bacterial species and CRP). IFNγ interferon-γ, TNF tumor necrosis factor; CRP C-reactive protein, TLR4 toll-like receptor 4, VEGF vascular endothelial growth factor, THBS1 thrombospondin 1, TM thrombomodulin.

We had previously probed the relationship between circulating plasma inflammatory cytokines and angiogenic protein levels, and disease characteristics^17–19^. We now tested if the microbial taxa identified as biomarkers of CA (Figs. 1 and 3) are associated with these circulating factors (Fig. 4a). Several correlations were observed between CA distinctive bacterial species and a number of circulating factors previously implicated in the disease (Fig. 4a). However, the weighted combined bacterial species best associated with disease activity had only a few significant correlations with plasma biomarkers, indicating that they might contribute complementary associations (Fig. 4b).

We hence tested whether microbiome abundance of distinctive species enhances disease severity association of plasma molecules. The best-weighted combination between *B. dorei* (sporadic/solitary vs. familial/multifocal) and LPB offered some improvement (Fig. 4c). The best-weighted combination between bacterial species identified in the aggressive CA patients and interleukin-10 (IL-10) significantly improved the ROC curve (area under the curve (AUC) = 0.90, Fig. 4d). This improvement was also seen when combining the species identified in CASH patients with plasma C-reactive protein (CRP) (AUC = 0.86, Fig. 4e). These results indicate that combining levels of circulating factors with bacterial species identified in clinically driven questions can enhance the disease severity association of either biomarker.

## Discussion

We show a diagnostic microbiome associated with high sensitivity and specificity with a human neurovascular disease, CA, predisposing to brain hemorrhage. This is linked to a defined mechanism whereby LPS produced by Gram-negative bacteria drives CA lesion genesis. The diagnostic microbiome is consistent among subjects recruited at different sites, and those harboring sporadic and familial disease and different CA genotypes. In humans as in mice, a permissive microbiome appears necessary for CA lesion development. We demonstrate other differences in microbiome and plasma molecules associated with disease severity features, and show how they can be combined for more precise associations with disease severity and hemorrhage.

Results herein represent a significant advance over previous pilot results demonstrating microbiome differences in CA subjects using 16S rRNA gene amplicon analysis^22^. Metagenomic shotgun sequencing allowed probing of specific bacterial species, sensitivity, and specificity determinations, network analyses and direct implication of their metabolic pathways. The expanded cohort allowed confirmation of prior 16S data in comparison to a different unrelated control population, with age and sex matching, and permitted replication of analyses of 16S data. Diagnostic microbiome was consistent regardless of genotype, gender, and at multiple sites of patient enrollment.

Our data strongly suggest a permissive microbiome related to postulated lesion genesis mechanisms, previously demonstrated in murine models^20^, rather than microbiome differences resulting from harboring a CA lesion. This is in view of (1) patients with familial/multifocal disease (with germline CCM mutations) and with sporadic/solitary lesions (without germline CCM gene mutations) have similar microbiome composition (Supplementary Fig. 2); (2) among familial/multifocal cases, those with greater CA lesion numbers had limited microbiome differences (Supplementary Fig. 4); and (3) bacterial species differences between sporadic/solitary and familial/multifocal disease, and between non-aggressive and aggressive CA patients are different from the permissive microbiome characterized between CA and non-CA patients. If harboring a CA were the cause of observed microbiome differences, we would expect the presence or absence of familial/multifocal disease with greater lesion numbers, and/or differences in disease aggressiveness would be associated with the same microbiome differences as CA vs. non-CA patients. These were not observed in our analyses.

Of the three species identified in the diagnostic microbiome (Fig. 1e, f), *F. prausnitzii* and *B. adolescentis* are Gram-positive species have been shown to be protective against gut inflammation^28–30^, and are less abundant in CA patients. The Gram-negative species *O. splanchnicus*, more abundant in CA patients, enhances gut inflammation, and has been linked to other neurological diseases^31^. Other species identified by multi-variate analysis are also largely protective against gut inflammation, with decreased abundance in CA patients. Based on such knowledge, our data suggest that CA patients have pathogenic microbiota, which may promote inflammation. Metagenomic shotgun sequencing-based pathway analysis and individual gene analyses suggest that CA-associated microbiome have increased LPS synthesis (Fig. 2a, b). Furthermore, we show decreased LPB concentrations in peripheral plasma of CA patients (Fig. 2c), consistent with increased LPS activity^25–27^.

Patients with aggressive vs. non-aggressive features of CA have significant differences of fecal microbiome, other than those identified in the diagnostic microbiome (Fig. 3, Supplementary Fig 4). This suggests that the microbiome differences favoring lesion genesis are not necessary or sufficient to drive disease severity. Microbiome organization differences with disease severity are evident by metagenomic shotgun sequencing-based co-occurrence network analyses and 16S rRNA gene amplicon sequencing-based α- and β-diversity analyses (Fig. 3a, e, Supplementary Fig. 4). Decreases in *Bacteroides* abundance were independently identified by both 16S rRNA gene amplicon sequencing and metagenomic shotgun sequencing approaches (Fig. 3b, f). Because the identified *Bacteroides* species have been shown to be protective in intestinal inflammation^32,33^, it is likely that loss of such *Bacteroides* species could contribute to the aggressive CA phenotype via altered intestinal mucosal homeostasis. Metagenomic shotgun sequencing identified species differences in cases with the aggressive vs. non-aggressive CA phenotype. One of the identified species, when comparing patients with non-aggressive and aggressive CA phenotype, *B. dorei*, is also the sole species differentiating CA patients with sporadic/solitary and familial/multifocal disease (Supplementary Fig. 2g). This species also negatively correlates with CA brain lesion counts in familial/multifocal patients (Supplementary Fig. 4d–f). Interestingly, *B. dorei* has been shown to be decreased in atherosclerosis patients. Introducing *B. dorei* to the gut of mice prone to have atherosclerosis decreases fecal LPS content and decreases atherosclerosis load, and *B. dorei* LPS is immunoinhibitory^32,33^. The exact contribution of *B. dorei* to CA disease needs to be further investigated in CA mouse models.

In the case of microbiome in CASH and non-CASH patients, no global microbiome differences were readily observed based on 16S rRNA gene amplicon sequencing. The metagenomic shotgun sequencing study showed both the Gram-positive species *F. prausnitzii* and Gram-negative *Oscillibacter* are both higher in CASH cases. *F. prausnitzii* is considered a protective bacterium for intestinal homeostasis and its decrease is part of the microbiome signature of CA patients. Lower *Oscillibacter* abundance has been associated with non-alcoholic liver disease and intestinal inflammation^34^. These suggest that development of CASH cannot be simply explained by an altered Gram-negative to Gram-positive bacteria ratio, or increases in pro-inflammatory bacteria. However, limited differences could also be explained by the low frequency of CASH cases in our cohort. Expanded patient numbers and longitudinal follow-up are needed to better investigate potential microbiome differences associated with lesional hemorrhage.

We have previously observed that CA patients have differences in circulating cytokines and other factors related to angiogenesis and inflammation^17–19^. We were able to detect direct associations between several CA distinctive bacterial species and plasma proteins implicated in disease activity (Fig. 4a, Supplementary Table 1). However, the best combined bacterial associations with CA diagnosis and disease severity had limited or no associations (Fig. 4b, Supplementary Table 1), indicating that the relationship between the microbiome and circulating factors may be complementary. Obtaining both plasma circulating factor and microbiome datasets from the same patients provided us an opportunity to explore the effectiveness of combining microbiome and circulating factors as biomarkers to differentiate CA with different characteristics (Fig. 4c–e). Our data suggest that microbiome and inflammation together drive CA disease severity, which may explain the improved ROC curve when combining microbiome signature of CA disease severity and pro-inflammatory cytokines. These proof-of-concept findings should motivate a larger prospective study, to validate combinations of microbiome and circulating factors as potential disease severity and prognostic biomarkers.

In summary, we have identified a pathogenic microbiome signature of human CA patients that is permissive for CA development, consistent with the previous mouse mechanistic studies demonstrating a role of LPS signaling. Different microbiome signatures are associated with disease severity features. Furthermore, we have demonstrated that application of machine learning methods can identify weighted combinations of microbiome signatures and plasma biomarkers that enhance disease severity associations. Future studies shall include larger cohorts with prospective follow-up, powered to detect associations in specific disease genotypes, sex, age, and disease activity. Laboratory, and ultimately translational research should also explore the impact of diet or specific microbiome modifications on CA development and bleeding. Approaches herein contribute to the understanding of a mechanistically defined neurovascular disease, and may be applicable to other diseases where gut–brain axis is postulated.

## Methods

### Stool collection and processing

Institutional review board approval, consistent with the Belmont Report and in compliance with the rules and regulations of the Federal Policy for the Protection of Human Subjects, was granted to all institutions, and all patients underwent written informed consent consistent with the Declaration of Helsinki. BSD/UCMC Institutional Review Boards at the University of Chicago, University of California San Francisco Institutional Review Board, University of New Mexico Institutional Review Board, and Quorum Review IRB (now part of Advarra) approved sample collections, at the University of Chicago, University of California San Francisco, University of New Mexico, and Angioma Alliance, respectively.

Stool collection was conducted by enrolling patients at clinic visits associated with routine CA medical care, or through recruiting phone calls. Stool samples were collected using in home stool collection kits (Thermo Fisher Scientific, Waltham, MA) and stored in at home refrigerators. Samples were then shipped to centralized sample collection center at the University of Pennsylvania via overnight shipment in insulated coolers with frozen ice packs. At the time of sample collection, patients also finished a survey, including major surgery history, antibiotic use, major gastrointestinal diseases, prebiotic and probiotic use, and other pertinent questions. Upon receipt, sample temperature was assessed and warm samples were rejected from further processing. Samples were then homogenized, aliquoted, and banked in −80 °C freezers until DNA exaction.

### Clinical parameters

Fecal samples were collected consecutively and prospectively from four institutions (The University of Chicago, Angioma Alliance, University of New Mexico and University of California San Francisco) between 10 April 2017 and 29 August 2018 (Supplementary Fig. 6). Inclusion criteria were a clinical index diagnosis of CA where at least one brain lesion had not been resected. CA disease severity features were collected from patients’ medical records^17^. The presence of inflammatory bowel disease and antibiotic exposure within 6 months were collected via the patient survey. Patient characteristics are detailed in Supplementary Table 2. MRI was completed on clinical 3-tesla scanners at participating sites. Susceptibility weighted sequences (SWI) and T2-weighted sequences (T2) were utilized to quantify lesion numbers. Lesions with a diameter > 4 mm on T2-weighted images and all lesions on SWI images were manually counted. Enrolled and screened patients had similar disease characteristics (Supplementary Table 3). Patients enrolled at the four sites had similar disease characteristics, with the exception of younger patients enrolled by the Angioma Alliance (Supplementary Table 4). Consistent with this, patients enrolled at all four sites have similar bacterial α-diversity (Supplementary Fig. 7). For 16S rRNA gene amplicon sequencing non-CA controls, patients within the publicly available American Gut Project Database^23^ were screened to generate age- and gender-matched controls free from neurological or other potential confounding parameters (*n* = 250, 128 females) with an average age of 42.5 (SD 18.3) Patients were excluded based on the following categories: outside the United States, Alzheimer’s, pregnant, phenylketonuria, substance abuse, bulimia nervosa, and anorexia nervosa. Metagenomic shotgun sequencing non-CA controls consist of stool samples banked at the University of Pennsylvania in a previously published study^35^ (*n* = 27, 17 female) with an age of 32.7 (SD 12.4) years. Potential age and gender confounders were adjusted in subsequent analyses.

All microbiome assessments were made by investigators blinded to any clinical features of the cases. Conversely, clinical features were adjudicated by the clinical investigative team prior to any microbiome evaluations.

### DNA purification

DNA was extracted from 200 mg of stool using the DNeasy PowerSoil Kit (Qiagen, Germantown, MD). Extracted DNA was quantified with the Quant-iT PicoGreen Assay Kit (Thermo Fisher).

### 16S rRNA gene amplicon sequencing

Libraries were generated using barcoded PCR primers 27F 5′-AGAGTTTGATCCTGGCTCAG-3′ and 338R 5′-TGCTGCCTCCCGTAGGAGT-3′ annealing to the V1–V2 region of the 16S rRNA gene^36^. PCR reactions were carried out in quadruplicate using Q5 High-Fidelity DNA Polymerase (New England Biolabs, Ipswich, MA). After amplification, quadruplicate PCR reactions were pooled and then purified using a 1:1 volume of SPRI beads (GE HealthCare, Chicago, IL). DNA in each sample was then quantified using Quant-iT PicoGreen Assay Kit (Thermo Fisher) and pooled in equal molar amounts. The resulting library was sequenced on the MiSeq instrument (Illumina, San Diego, CA) using 2 × 250 bp chemistry. Extraction blanks and DNA-free water were subjected to the same amplification and purification procedure to allow for empirical assessment of environmental and reagent contamination. Positive controls, consisting of eight artificial 16S gene fragments synthesized in gene blocks and combined in known abundances, were also included^37^.

### Metagenomic shotgun sequencing

Shotgun libraries were generated using the NexteraXT Kit (Illumina) and sequenced on the HiSeq 2500 instrument (Illumina) using 2 × 125 bp chemistry. Extraction blanks and DNA-free water were processed the same way as the samples and were included to empirically assess environmental and reagent contamination. Laboratory-generated mock communities consisting of DNA from *Vibrio campbellii* and Lambda phage were included as positive controls.

### 16S rRNA gene amplicon sequencing analyses

16S rRNA gene amplicon sequencing data from enrolled CA patients and American Gut Project non-CA were quality-filtered and de-multiplexed using QIIME 2^38^. Because different regions were sequenced for enrolled CA patients (V1–V2) and American Gut project (V4), fragment insertion methodology (also known as SEPP) was used in QIIME 2 to overcome the potential bias for sequencing different regions of the 16S rRNA gene. De-multiplexed sequences were then selected for ESV picking using DeBlur trimmed to 125 nucleotides^39^. ESVs present in <10 samples were removed using Phyloseq. The final BIOM file comprising of unique 60,141 ESVs with average 32,190 reads per sample was then used for further analyses. Stool samples with low read counts were excluded from analysis. For five patients with two separate stool sample collections, they are considered as one sample during analysis. Richness, Shannon, and Simpson indices as measurements of α-diversity were calculated and compared using phyloseq package in R. Variations between groups (β-diversity) was statistically tested using permutational multi-variate analysis of variance (PERMANOVA) using microbiomeSeq package in R. Analysis of composition of microbiomes (ANCOM) was used to identify differentially abundant bacterial ESVs between the groups at *p* value cut-off of 0.05 with Benjamini–Hochberg FDR correction^40^. The confounding variable, that is, age, gender, and collection site were adjusted for in the ANCOM analyses. For gender-specific studies, ANCOM analyses were adjusted for age. ANCOM results were plotted using box plots in R, with the boxes representing the interquartile range for the data, the line in the middle of the box is the median, the whiskers are the minimum and maximum of the values in the data, and the star is the mean of the data. The + signs are the outliers. Spearman’s rank correlation and generalized linear models (GLMs) were used to establish association between the microbiome and other continuous variables in the metadata using microbiomeSeq() and glm() packages in R.

#### Control for batch-to-batch variations and duplicate validation

16S rRNA gene amplicon sequencing was completed in two batches, including a repeated run of 40 randomly selected samples for duplicated validation. To limit run-to-run influence, 40 samples, randomized by the category from batch 1 were re-run in batch 2, providing overlapping duplicates between runs. Some runs were removed from analysis due to duplicated sample collection of the same patient, and samples received at incorrect temperatures. The Shannon α-diversity values and β-diversity indices (weighted UniFrac) were then compared for the overlapping samples between two runs. PERMANOVA was used and re-run sequencing results were not significantly different relative to the initial run (weighted UniFrac distance ≤ 0.06 in all cases; p_PERMANOVA_ > 0.05). For samples sequenced in both batch one and batch two, batch two data was used for subsequent analyses.

#### Analysis with two control groups

Although differences in sequencing technology between the CA patients and American Gut Project were minimized as much as possible using established protocols, it is still possible that methodological differences in the 16S rRNA gene amplicon sequencing of American Gut Project control samples could introduce bias and lead to apparent differences in the microbiome. To address this concern, a second control sample set was used. This sample set was chosen from banked stool samples from the University of Pennsylvania^35^. β-Diversity analysis showed that although separation between CA patients and this control cohort was less pronounced, it was still significant (Supplementary Fig. 1b), despite a smaller number of samples in this control group. This result increases confidence that the microbiota of the CA cohort are different relative to two distinct non-CA cohorts. This notion was further supported by metagenomic sequencing analysis results.

### Metagenomic shotgun sequencing analysis

For metagenomic shotgun sequencing analysis, 2.2 billion paired-end metagenome reads were quality trimmed (for adapters, primers, and oligonucleotides) with Nesoni. To assess taxonomic diversity, trimmed data were analyzed using MetaPhlAn2 to profile the composition of microbial communities (bacteria, archaea, eukaryotes, and viruses) at the species level^41^. A database of ~1 M unique clade-specific marker genes identified from ~17,000 reference genomes were used in MetaPhlAn2, and BowTie2 was used for reference-based alignment of the reads^42^. For five patients with two separate stool sample collections, they are considered as one sample during analysis. Co-occurrence networks were generated by calculating Spearman’s correlations between abundance of species using Hmisc in R. Significant connections (Benjamini–Hochberg FDR-corrected *p* value < 0.05) were exported as GML format network files using igraph in R. The modularity analyses and keystone node identification were performed using Gephi. ANCOM was used to identify differentially abundant bacterial species between the groups at *p* value cut-off of 0.05 with Benjamini–Hochberg FDR correction^40^. Confounding variables, including age, gender, and collection site, were adjusted for in ANCOM analyses. For gender-specific studies, ANCOM analyses were adjusted for age. Functional profiling was performed using HUMAnN2, which identifies the species profile from metagenomic shotgun sequencing data and aligns reads to their pangenomes, performs translated search on unclassified reads, and quantifies gene families and pathways^43^. HUMAnN2 was used to regroup gene families to MetaCyc reactions. Differential analyses and plotting was performed using ANCOM. Age, gender, and sites were adjusted for in all the analyses. Random forest algorithm was used to extract the most important features in the microbiome species information using the Boruta pipeline^44^. Spearman’s rank correlation and GLMs were used to establish association between the microbiome and other continuous variables in the metadata using microbiomeSeq() and glm() packages in R. For control population, a control cohort stool samples banked at the University of Pennsylvania Microbiome Center were processed^35^, sequenced, and analyzed along with CA cohort samples, and sequencing results were corrected for age and gender during analysis.

### Plasma biomarker measurements

Whole blood was collected in a subset of patients (*n* = 47) from the University of Chicago site^17–19^. Plasma were separated from cellular components of the blood, aliquoted, and stored at −80 °C until analysis. Quantification of plasma biomarkers were performed using commercially available individual and multiplex ELISA Kits. ELISA assays were performed according the manufacturer’s protocols. For individual ELISA assays (TLR4 [Raybiotech ELHTLR41], LPB [R&D Systems, DY87005], CRP[R&D Systems, DCRP00], sCD14 [R&D Systems, DC140], endoglin [R&D Systems, DNDG00], thrombospondin 1 [R&D Systems, DTHBD0], thrombomodulin [R&D Systems, DTSP10]), plates were washed using a BioTek 405TS automatic plate washer (BioTek Instruments, Winooski, VT, USA), and absorbance were measured using a Bio-Rad iMark plate reader (Bio-Rad, Hercules, CA, USA). Multiplex ELISA assays (interferon-γ, IL-10, IL-1β, IL-6, tumor necrosis factor, vascular endothelial growth factor) were performed using a V-plex multi-spot assay and detection system (Meso Scale Diagnostics, Rockville, MD, USA). LBP and other individually measured plasma factors were performed in two batches, and batch effect was corrected by applying Bioconductor package limma (https://bioconductor.org/packages/release/bioc/html/limma.html)^45^, and was compared by unpaired *t* test with Welch’s correction. Samples with values outside 2 SD of mean were excluded from the analysis.

### Integrated analyses of microbiome and plasma data

Association of relative abundance of metagenomic shotgun sequencing determined species and plasma biomarkers was determined by calculating the Pearson’s correlation coefficient with Benjamini–Hochberg FDR correction. Plasma marker values 2 SD from mean are excluded from the analysis. To evaluate the clinical predictive performance of metagenomic shotgun sequencing determined species, plasma biomarkers, and combination of microbial species and plasma biomarkers to differentiate CA patients with distinctive disease characteristics, logistic models were used. Linear discriminant analyses were conducted to acquire combined scores. AUC and 95% confidence interval from the calculated ROC curve. Optimal values of specificity and sensitivity were determined by Youden index to measure the best fit of a model. Analyses were performed using SAS 9.4 (SAS, Cary, NC).

### Reporting summary

Further information on research design is available in the Nature Research Reporting Summary linked to this article.

## Supplementary information

Supplementary Information

Reporting Summary

## Data Availability

16S sequencing dataset is deposited into European Molecular Biology Laboratory-European Nucleotide Archive EMBL-ENA with a project ID PRJEB35505 [https://www.ebi.ac.uk/ena/data/view/PRJEB35505]. Metagenomic shotgun sequencing dataset is deposited into Sequence Read Archive at National Center for Biotechnology Information with a Bioproject ID PRJNA629755 [https://www.ncbi.nlm.nih.gov/bioproject/PRJNA629755]. Data underlying all figures are provided as Source data files. Other information is available from corresponding authors upon reasonable requests.

## Source data

Source Data
