# Supplementary material for: Permissive microbiome characterizes human subjects with a neurovascular disease cavernous angioma
Source: Nat Commun. 2020 May 27;11:2659. doi: 10.1038/s41467-020-16436-w (PMC7253448; doi:10.1038/s41467-020-16436-w)
Supplement: Supplementary file 1 — Supplementary Information [file 41467_2020_16436_MOESM1_ESM.pdf]

Supplementary Information

Polster and Sharma *et al.*

Permissive microbiome characterizes human subjects with a neurovascular disease cavernous angioma

Supplementary Discussion

The 16S rRNA gene amplicon sequencing data was consistent when repeated at two points in time in 38 randomly selected specimens, indicating no batch effect of sequencing. Furthermore, there was no difference related to the clinical sites (Suppl. Fig. 7). To test if including all overlapping bacterial species, identified by both multi-variate and random forest analysis with a relaxed medium relative abundance and significance cutoff (medium relative abundance ≥0.1%, FDR corrected p ≤0.05), could improve discriminative power, ROC curve was generated by using the best combination of all nine overlapping species (Suppl. Fig. 1F). The ROC curve had moderately improved AUC (0.892 vs 0.826) relative to three species with stringent medium relative abundance and significance cutoff.

Because CA patients can have either sporadic/solitary or familial/multifocal form of the disease (Suppl. Fig. 2A), and only familial/multifocal group has germline *CCM* gene mutations, it is possible that mixing sporadic/solitary and familial/multifocal CA patients in our analysis could obscure microbiome differences in either cohort. 16S rRNA gene amplicon sequencing studies showed no difference in α diversity (Suppl. Fig. 2B), but with significant changes in four multi-variate identified ESVs (Suppl. Fig. 2C). Co-occurrence network studies based on metagenomic shotgun sequencing data showed that sporadic/solitary and familial/multifocal CA patients have different microbial network organization with distinct keystone species (Suppl. Fig. 2D). At the species level, analysis of metagenomic shotgun data show many species are similarly changed in both sporadic/solitary and familial/multifocal CA patients (Suppl. Fig. 2D, E), suggesting that combining sporadic/solitary and familial/multifocal CA patients does not mask differences in either group. A single *Bacteroides dorei* species was identified to be more abundant in sporadic/solitary patients than both non-CA individuals and familial/multifocal patients (Suppl. Fig. 2F, G); however, it was not able to differentiate sporadic/solitary and familial/multifocal CA patients effectively (Suppl. Fig. 2E). These data suggest that CA patients with both sporadic/solitary and familial/multifocal disease have different microbiota than non-CA patients, while differences between sporadic/solitary and familial/multifocal CA patients are limited.

Our data showed that the fecal microbiome in patients with or without germline *CCM* mutations have different organization, (Suppl. Fig. 2B, D). However, very limited species difference was observed in patients with sporadic lesions (without *CCM* germ line mutations) as compared to cases with familial multifocal disease (harboring *CCM* germ line mutations). It is also possible that mutations of different *CCM* genes impact microbiome in different manners, thus combining data from patients with mutations of

different *CCM* genes could obscure the difference between sporadic/solitary and familial/multifocal CA patients. However, metagenomic shotgun studies failed to show significant differences between CA patients with mutations of different *CCM* genes, while some differences can be identified by 16S rRNA gene amplicon sequencing (Suppl. Fig. 3). This calls for future studies with a larger number of CA patients with mutations of different *CCM* genes. Although some fecal microbiome differences can be observed between CASH and non-CASH patients, they do not support effective differentiation between these two CA patient groups (Fig. 3). This may be partially caused by the low CASH case enrollment in the current study. To properly address this question a higher number of cases with symptomatic hemorrhage will be required.

We have adjusted our analyses based on age, gender, and collection site so they do not confound our analyses by excluding species with significant age, gender, and collection site differences. Here, we specifically asked if female and male CA patients have different microbiome and if female CA patients and male CA patients are differentially affected relative to female non-CA and male non-CA patients, respectively. We compared 16S rRNA gene amplicon and metagenomic shotgun sequencing data by multi-variate analyses. Differences between female and male non-CA patients and between female and male CA patients were determined by multi-variate analysis. Relative abundance of these taxonomy units, within these four patient groups, were then compared. Some differences can be seen (Suppl. Fig. 5C, D). With the exception of the low abundance Barnesiellaceae family (Suppl. Fig. 5A) and *Bacteriodes fragilis* (Suppl. Fig. 5B), these species were not differentially represented in female and male CA patients relative to female and male non-CA individuals respectively. Taken together, these results do not support a drastically different microbiome modulation in female non-CA and CA patients relative to male non-CA and CA patients. Because the most significant microbiome differences within the CA cohort was identified in patients with or without aggressive CA disease, we further compared microbiota between female and male CA patients with aggressive disease and between female and male non-aggressive CA patients by multi-variate analysis. Relative abundances of these taxonomy units within these four patient groups were then compared. Some differences can be seen (Suppl. Fig. 5C, D). With the exception of the low abundance S24-7 family (Suppl. Fig. 5C) and *Eubacterium siraenum* (Suppl. Fig. 5D), these species were not differentially represented in female and male patients with aggressive CA relative to female and male patients with non-aggressive CA, respectively. Taken together, these results do not support a drastically different microbiome modulation in female non-aggressive and aggressive CA patients relative to male non-aggressive and aggressive CA patients.

Supplementary Figures

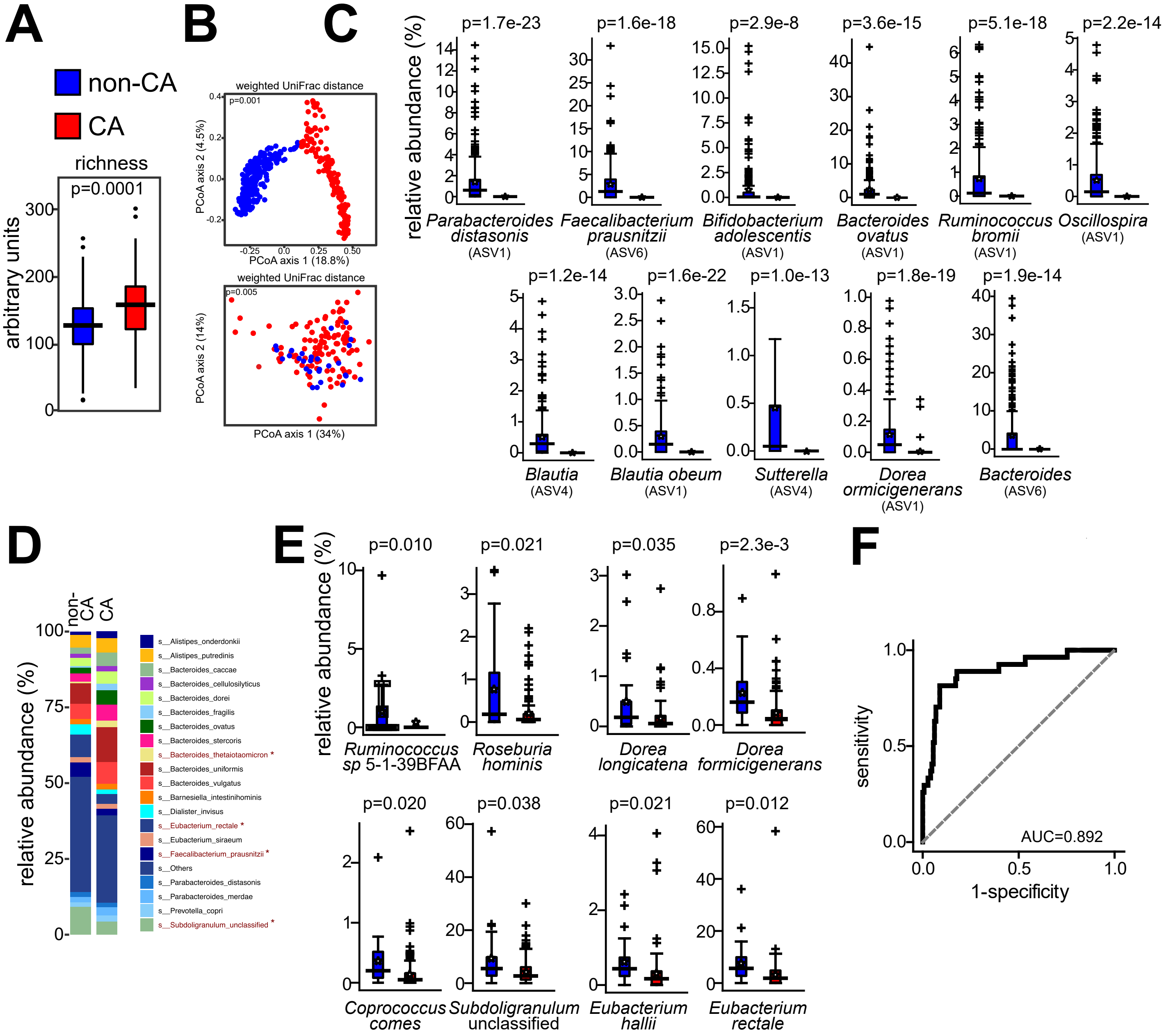
A
B
C
non-CA
CA
richness
p=0.0001
arbitrary units
weighted UniFrac distance
p=0.001
PCoA axis 1 (18.8%)
PCoA axis 2 (4.5%)
weighted UniFrac distance
p=0.005
PCoA axis 1 (34%)
PCoA axis 2 (14%)
relative abundance (%)
p=1.7e-23
Parabacteroides distasonis (ASV1)
p=1.6e-18
Faecalibacterium prausnitzii (ASV6)
p=2.9e-8
Bifidobacterium adolescentis (ASV1)
p=3.6e-15
Bacteroides ovatus (ASV1)
p=5.1e-18
Ruminococcus bromii (ASV1)
p=2.2e-14
Oscillospira (ASV1)
p=1.2e-14
Blautia (ASV4)
p=1.6e-22
Blautia obeum (ASV1)
p=1.0e-13
Sutterella (ASV4)
p=1.8e-19
Dorea ormicigenerans (ASV1)
p=1.9e-14
Bacteroides (ASV6)
D
non-CA
CA
relative abundance (%)
s__Alistipes_onderdonkii
s__Alistipes_putredinis
s__Bacteroides_caccae
s__Bacteroides_cellulosilyticus
s__Bacteroides_dorei
s__Bacteroides_fragilis
s__Bacteroides_ovatus
s__Bacteroides_stercoris
s__Bacteroides_thetaiotaomicron *
s__Bacteroides_uniformis
s__Bacteroides_vulgatus
s__Barnesiella_intestinihominis
s__Dialister_invisus
s__Eubacterium_rectale *
s__Eubacterium_siraeum
s__Faecalibacterium_prausnitzii *
s__Others
s__Parabacteroides_distasonis
s__Parabacteroides_merdae
s__Prevotella_copri
s__Subdoligranulum_unclassified *
E
relative abundance (%)
p=0.010
Ruminococcus sp 5-1-39BFAA
p=0.021
Roseburia hominis
p=0.035
Dorea longicatena
p=2.3e-3
Dorea formicigenerans
p=0.020
Coprococcus comes
p=0.038
Subdoligranulum unclassified
p=0.021
Eubacterium hallii
p=0.012
Eubacterium rectale
F
sensitivity
1-specificity
AUC=0.892

Supplementary Figure 1. CA and non-CA cohorts have different microbiota.

A. α diversity analyses of fecal samples by richness index based on 16S rRNA gene amplicon sequencing data (n=250 non-CA, n=115 CA, Kruskal-Wallis one way analysis of variance test, blue boxes: non-CA cohort, red boxes: CA cohort). **B.** PCoA analysis of β diversity of fecal samples based on 16S rRNA gene amplicon sequencing data. Top panel: comparing with American Gut Project control samples (PERMANOVA test, p=0.001); bottom panel: comparing with University of Pennsylvania control samples (PERMANOVA test, p=0.005). blue: non-CA cohort, red: CA cohort. **C.** Multi-variate taxonomic analyses of 16S rRNA gene amplicon sequencing data. ESVs with significantly different relative abundances (n=250 non-CA, n=115 CA, ANCOM analysis followed by two-sided Mann-Whitney U test with Benjamini-Hochberg FDR correction, $p_{FDR} \leq 0.05$ but >0.01) or medium relative abundance of <1% in both groups are presented as box-whisker plots (blue boxes: non-CA cohort, red boxes: CA cohort). **D.** Top 20 most abundant species identified based on metagenomic shotgun sequencing data. Species with significantly different abundance are presented in maroon color. **E.** Multi-variate analyses of metagenomic shotgun sequencing data at species level. Species with significantly different abundance (n=27 non-CA, n=122 CA, ANCOM analysis followed by two-sided Mann-Whitney U test with Benjamini-Hochberg FDR correction, $p_{FDR} \leq 0.05$, but >0.01) or medium relative abundance <0.25% in both groups are presented as box-whisker plots (blue boxes: non-CA cohort, red boxes: CA cohort). **F.** ROC curve was identified based on best-weighted combination of all nine common bacterial species (medium relative abundance ≥0.1% in either groups, p ≤0.05) identified by multi-variate and random forest analysis (AUC=0.892, specificity=0.814, sensitivity=0.911). In box plots, bounds of boxes show IQR, top and bottom whiskers demonstrate maxinum and mininum, lines in the middle of the box indicate median, and stars show mean of the data. + signs indicate outliers.

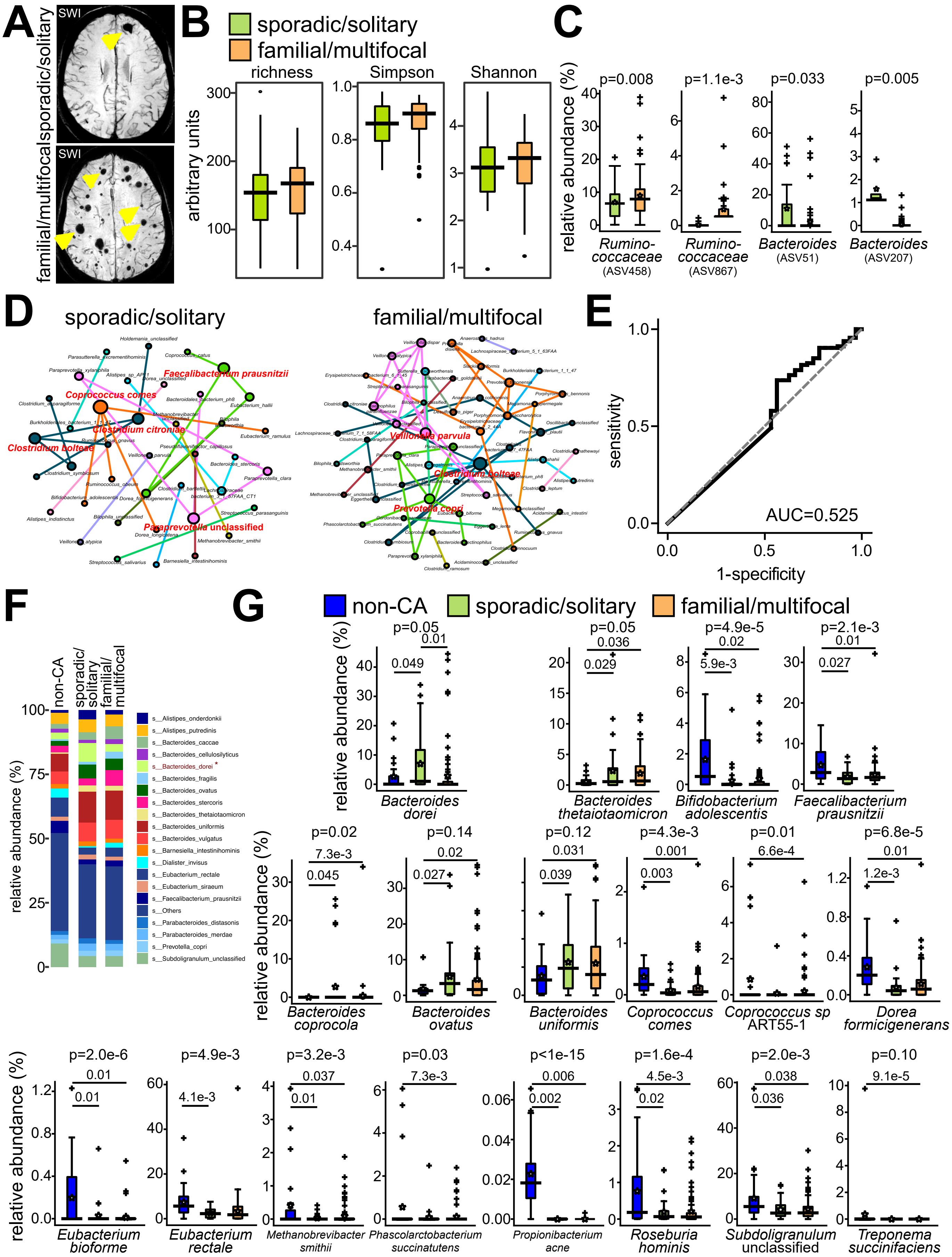

A
SWI
sporadic/solitary
familial/multifocal
B
sporadic/solitary
familial/multifocal
richness
Simpson
Shannon
arbitrary units
C
relative abundance (%)
p=0.008
p=1.1e-3
p=0.033
p=0.005
Rumino-coccaceae (ASV458)
Rumino-coccaceae (ASV867)
Bacteroides (ASV51)
Bacteroides (ASV207)
D
sporadic/solitary
familial/multifocal
Faecalibacterium prausnitzii
Coprococcus comes
Clostridium citroniae
Clostridium bolteae
Paraprevotella unclassified
Veillonella parvula
Clostridium bolteae
Prevotella copri
E
sensitivity
1-specificity
AUC=0.525
F
non-CA
sporadic/solitary
familial/multifocal
relative abundance (%)
s__Alistipes_onderdonkii
s__Alistipes_putredinis
s__Bacteroides_caccae
s__Bacteroides_cellulosilyticus
s__Bacteroides_dorei *
s__Bacteroides_fragilis
s__Bacteroides_ovatus
s__Bacteroides_stercoris
s__Bacteroides_thetaiotaomicron
s__Bacteroides_uniformis
s__Bacteroides_vulgatus
s__Barnesiella_intestinihominis
s__Dialister_invisus
s__Eubacterium_rectale
s__Eubacterium_siraeum
s__Faecalibacterium_prausnitzii
s__Others
s__Parabacteroides_distasonis
s__Parabacteroides_merdae
s__Prevotella_copri
s__Subdoligranulum_unclassified
G
non-CA
sporadic/solitary
familial/multifocal
relative abundance (%)
p=0.05
0.01
0.049
Bacteroides dorei
p=0.05
0.036
0.029
Bacteroides thetaiotaomicron
p=4.9e-5
0.02
5.9e-3
Bifidobacterium adolescentis
p=2.1e-3
0.01
0.027
Faecalibacterium prausnitzii
p=0.02
7.3e-3
0.045
Bacteroides coprocola
p=0.14
0.02
0.027
Bacteroides ovatus
p=0.12
0.031
0.039
Bacteroides uniformis
p=4.3e-3
0.001
0.003
Coprococcus comes
p=0.01
6.6e-4
Coprococcus sp ART55-1
p=6.8e-5
0.01
1.2e-3
Dorea formicigenerans
p=2.0e-6
0.01
0.01
Eubacterium bioforme
p=4.9e-3
4.1e-3
Eubacterium rectale
p=3.2e-3
0.037
0.01
Methanobrevibacter smithii
p=0.03
7.3e-3
Phascolarctobacterium succinatutens
p<1e-15
0.006
0.002
Propionibacterium acne
p=1.6e-4
4.5e-3
0.02
Roseburia hominis
p=2.0e-3
0.038
0.036
Subdoligranulum unclassified
p=0.10
9.1e-5
Treponema succinifaciens

Supplementary Figure 2. CA patients with or without *CCM* germline mutations.

A. MRI images of CA patients. Susceptibility weighted imaging (SWI) showing a sporadic/solitary CA patient with a left frontal CA (left panel) and a familial/multifocal CA patient with >100 different sized lesions throughout the brain (right panel). CA lesions are indicated by yellow arrows. **B.** α diversity analyses of fecal samples by richness, Shannon and Simpson indices based on 16S rRNA gene amplicon sequencing data, presented as box-whisker plots (n=28 sporadic/solitary, n=87 familial/multifocal, Kruskal-Wallis one way analysis of variance test, blue boxes: sporadic/solitary patients, red boxes: familial/multifocal patients). **C.** Multi-variate taxonomic analyses at ESV level between sporadic/solitary and familial/multifocal CA patients. ESVs with significantly different relative abundances are presented as box-whisker plots (n=28 sporadic/solitary, n=87 familial/multifocal, ANCOM analysis followed by two-sided Mann-Whitney U test with Benjamini-Hochberg FDR correction, blue boxes: sporadic/solitary patients, red boxes: familial/multifocal patients). **D.** Organization of microbiome species in sporadic/solitary and familial/multifocal cohorts. Co-occurrence network analyses were performed at species level, as determined by metagenomic shotgun sequencing data analysis (n=29 sporadic, n=93 familial, keystone species are labeled in red). orange: CA patients with familial/multifocal disease). **E.** ROC model for *B. dorei* to differentiate sporadic/solitary and familial/multifocal CA patients (AUC=0.514. specificity=0.438, sensitivity=0.716). **F.** Top 20 most abundant species identified by metagenomic shotgun sequencing data analysis among non-CA, CA patients with sporadic/solitary disease, and familial/multifocal disease. The species with significantly different abundance is presented in maroon color. **G.** Multigroup analysis of metagenomic shotgun sequencing data among non-CA, sporadic/solitary, and familial/multifocal patients at species level. Species with significantly different abundance are presented as box-whisker plots (n=28 sporadic/solitary, n=87 familial/multifocal, ANCOM analysis followed by two-sided Mann-Whitney U test with Benjamini-Hochberg FDR correction, blue: non-CA individuals, green: CA patients with sporadic/solitary disease, orange: CA patients with familial/multifocal disease). P values of *post hoc* analysis identified significant differences are shown. In box plots, bounds of boxes show IQR, top and bottom whiskers demonstrate maxinum and mininum, lines in the middle of the box indicate median, and stars show mean of the data. + signs indicate outliers.

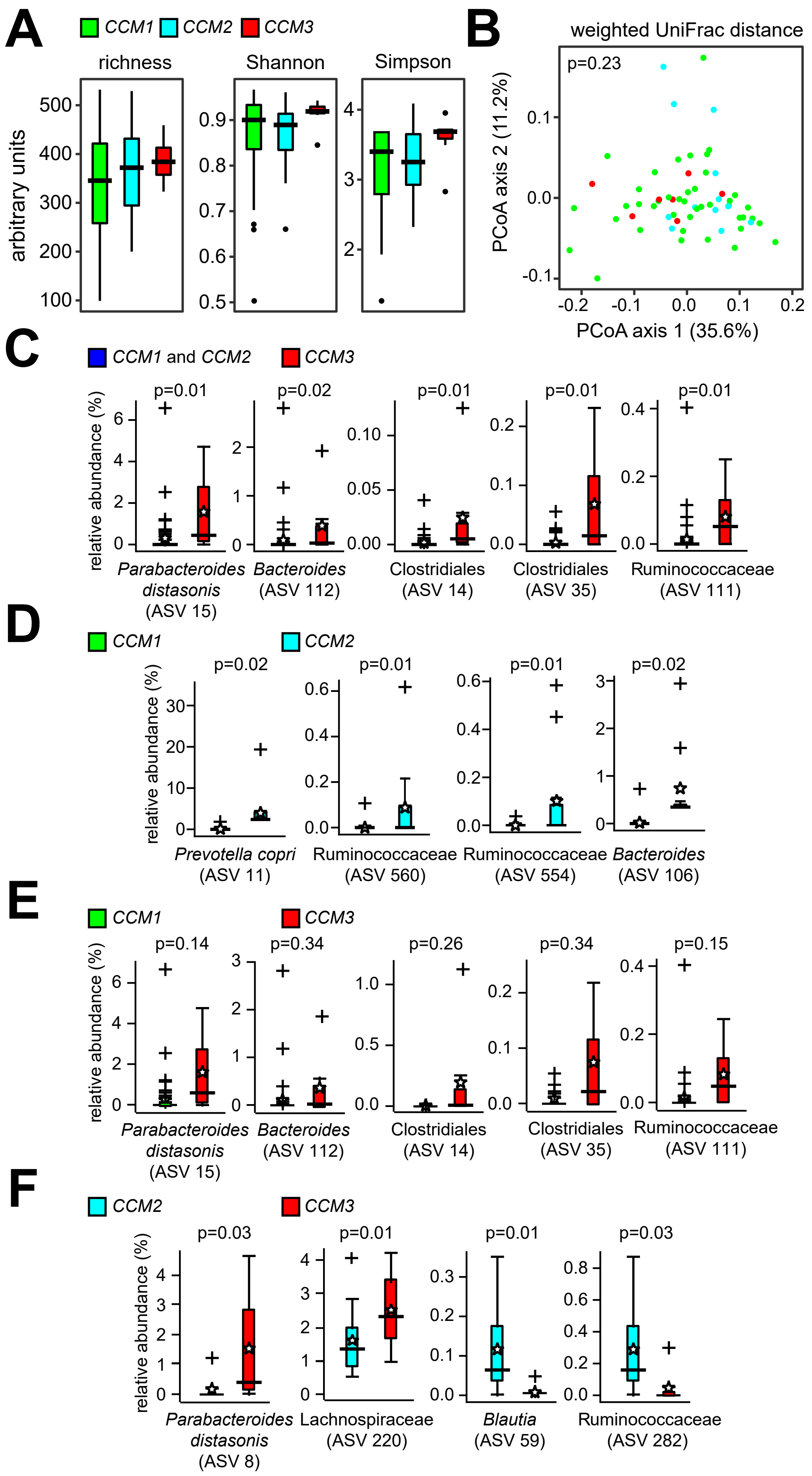
A
CCM1 CCM2 CCM3
richness
Shannon
Simpson
arbitrary units
B
weighted UniFrac distance
p=0.23
PCoA axis 2 (11.2%)
PCoA axis 1 (35.6%)
C
CCM1 and CCM2
CCM3
p=0.01
p=0.02
p=0.01
p=0.01
p=0.01
relative abundance (%)
Parabacteroides distasonis (ASV 15)
Bacteroides (ASV 112)
Clostridiales (ASV 14)
Clostridiales (ASV 35)
Ruminococcaceae (ASV 111)
D
CCM1
CCM2
p=0.02
p=0.01
p=0.01
p=0.02
relative abundance (%)
Prevotella copri (ASV 11)
Ruminococcaceae (ASV 560)
Ruminococcaceae (ASV 554)
Bacteroides (ASV 106)
E
CCM1
CCM3
p=0.14
p=0.34
p=0.26
p=0.34
p=0.15
relative abundance (%)
Parabacteroides distasonis (ASV 15)
Bacteroides (ASV 112)
Clostridiales (ASV 14)
Clostridiales (ASV 35)
Ruminococcaceae (ASV 111)
F
CCM2
CCM3
p=0.03
p=0.01
p=0.01
p=0.03
relative abundance (%)
Parabacteroides distasonis (ASV 8)
Lachnospiraceae (ASV 220)
Blautia (ASV 59)
Ruminococcaceae (ASV 282)

Supplementary Figure 3. CA patients with *CCM1*, *CCM2*, and *CCM3* mutations.

16S rRNA gene amplicon sequencing data was used to determine microbiome differences in CA patients with mutations of different *CCM* genes. **A.** α diversity analysis of fecal microbiome of CA patients with *CCM1* (n=43, green), *CCM2* (n=12, cyan), and *CCM3* (n=7, red) mutations (Kruskal-Wallis one way analysis of variance test). **B.** PCoA plot for β diversity analysis of fecal microbiome of CA patients with *CCM1* (green), *CCM2* (cyan), and *CCM3* (red) mutations (PERMANOVA test). **C.** Patients with *CCM1* or *CCM2* mutations (n= 55, blue) relative to patients with *CCM3* mutations (n=7, red, ANCOM analysis followed by two-sided Mann-Whitney U test with Benjamini-Hochberg FDR correction). **D.** Patients with *CCM1* mutations (n=43, green) relative to patients with *CCM2* mutations (n=12, cyan, ANCOM analysis followed by two-sided Mann-Whitney U test with Benjamini-Hochberg FDR correction). **E.** Patients with *CCM1* (n=43, green) relative to patients with *CCM3* mutations (n=7 red, ANCOM analysis followed by two-sided Mann-Whitney U test with Benjamini-Hochberg FDR correction). Please note none of the listed ESVs in this panel is statistically significant. **F.** Patients with *CCM2* mutations (n=12, cyan) relative to patients with *CCM3* mutations (n=7, red, ANCOM analysis followed by two-sided Mann-Whitney U test with Benjamini-Hochberg FDR correction). In box plots, bounds of boxes show IQR, top and bottom whiskers demonstrate maxinum and mininum, lines in the middle of the box indicate median, and stars show mean of the data. + signs indicate outliers.

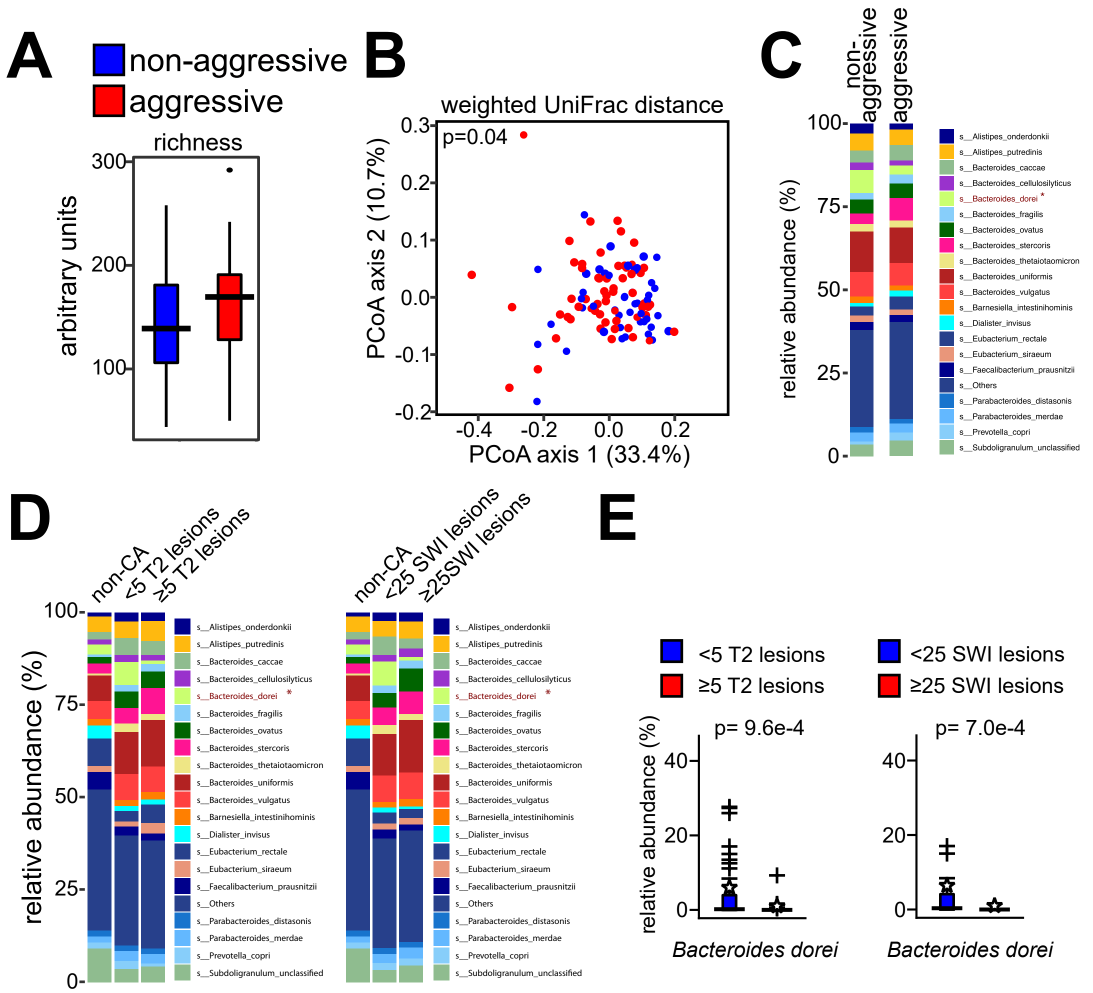

F

Taxa	Continuous Variable	Correlation	p (FDR corrected)
Bacteroides dorei	Number_SWI	-0.32	0.0013
Bacteroides dorei	Number_T2	-0.24	0.012

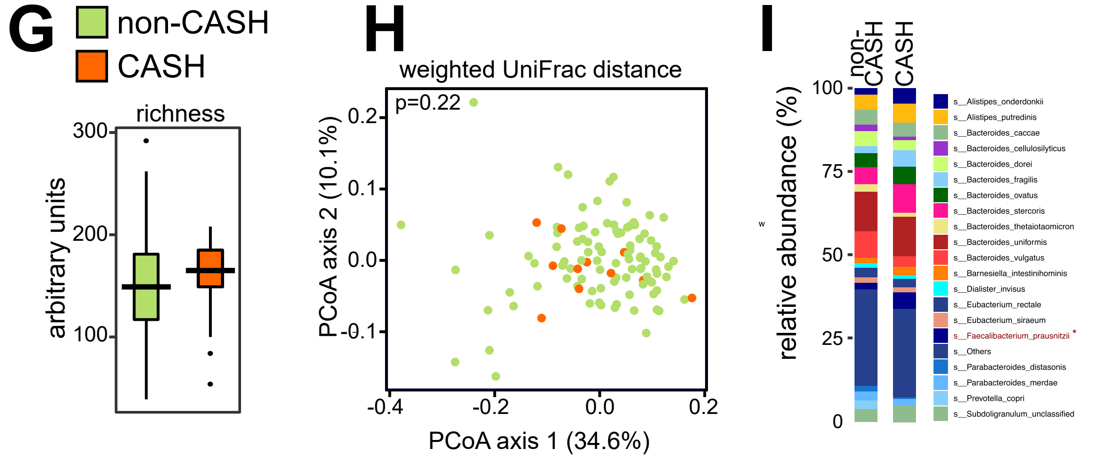

Supplementary Figure 4. CA patients with different disease properties.

A. α diversity analyses of fecal samples of CA patients with non-aggressive and aggressive disease by richness index based on 16S rRNA gene amplicon sequencing data (n=43 non-aggressive patients, n=58 aggressive patients, Kruskal-Wallis one way analysis of variance test, blue box: non-aggressive CA patients, red box: aggressive CA patients). **B.** PCoA analysis of β diversity of fecal samples based on16S rRNA gene amplicon sequencing data of CA patients with non-aggressive and aggressive disease (n=43 non-aggressive patients, n=58 aggressive patients, PERMANOVA test, blue: non-aggressive CA patients, red: aggressive CA patients). **C.** Top 20 most abundant species identified based on metagenomic shotgun sequencing data. Species with significantly different abundance are presented in maroon color. **D.** Top 20 most abundant species identified by metagenomic shotgun sequencing data analysis between familial/multifocal patients with or without high CA lesion counts identified by either T2-weighted or susceptibility weighted imaging (SWI) MRI imaging modules. The species with significantly different abundance is presented in maroon color. **E.** Multi-variate analysis of metagenomic shotgun sequencing data between familial/multifocal patients with or without high CA lesion counts identified by either T2-weighted (n=44 for low lesion count, n=28 for high lesion counts, ANCOM analysis followed by two-sided Mann-Whitney U test with Benjamini-Hochberg FDR correction, blue boxes: low lesion counts, red boxes: high lesion counts) or SWI (n=37 for low lesion count, n=28 for high lesion counts, ANCOM analysis followed by two-sided Mann-Whitney U test with Benjamini-Hochberg FDR correction, blue boxes: low lesion counts, red boxes: high lesion counts) MRI imaging modules. Species with significantly different abundance are presented as box-whisker plots. **F.** Negative correlation of T2-weighted (n=65) or SWI (n=72) lesion number in familial/multifocal patients with relative abundance of *B. dorei* (Spearman correlation and GLM test with Benjamini-Hochberg FDR correction). **G.** α diversity analyses of fecal samples of CA patients with non-CASH and CASH disease by richness index based on 16S rRNA gene amplicon sequencing data (n=93 non-CASH patients, n=13 CASH patients, PERMANOVA test, green box: non-CASH CA patients, orange box: CASH CA patients). **H.** PCoA analysis of β diversity of fecal samples based on16S rRNA gene amplicon sequencing data of CA patients with non-CASH and CASH disease (n=93 non-CASH patients, n=13 CASH patients, PERMANOVA test, green: non-CASH CA patients, orange: CASH CA patients). **I.** Top 20 most abundant species identified based on metagenomic shotgun sequencing data. Species with significantly different abundance are presented in maroon color. In box plots, bounds of boxes show IQR, top and bottom whiskers demonstrate maxinum and mininum, lines in the middle of the box indicate median, and stars show mean of the data. + signs indicate outliers.

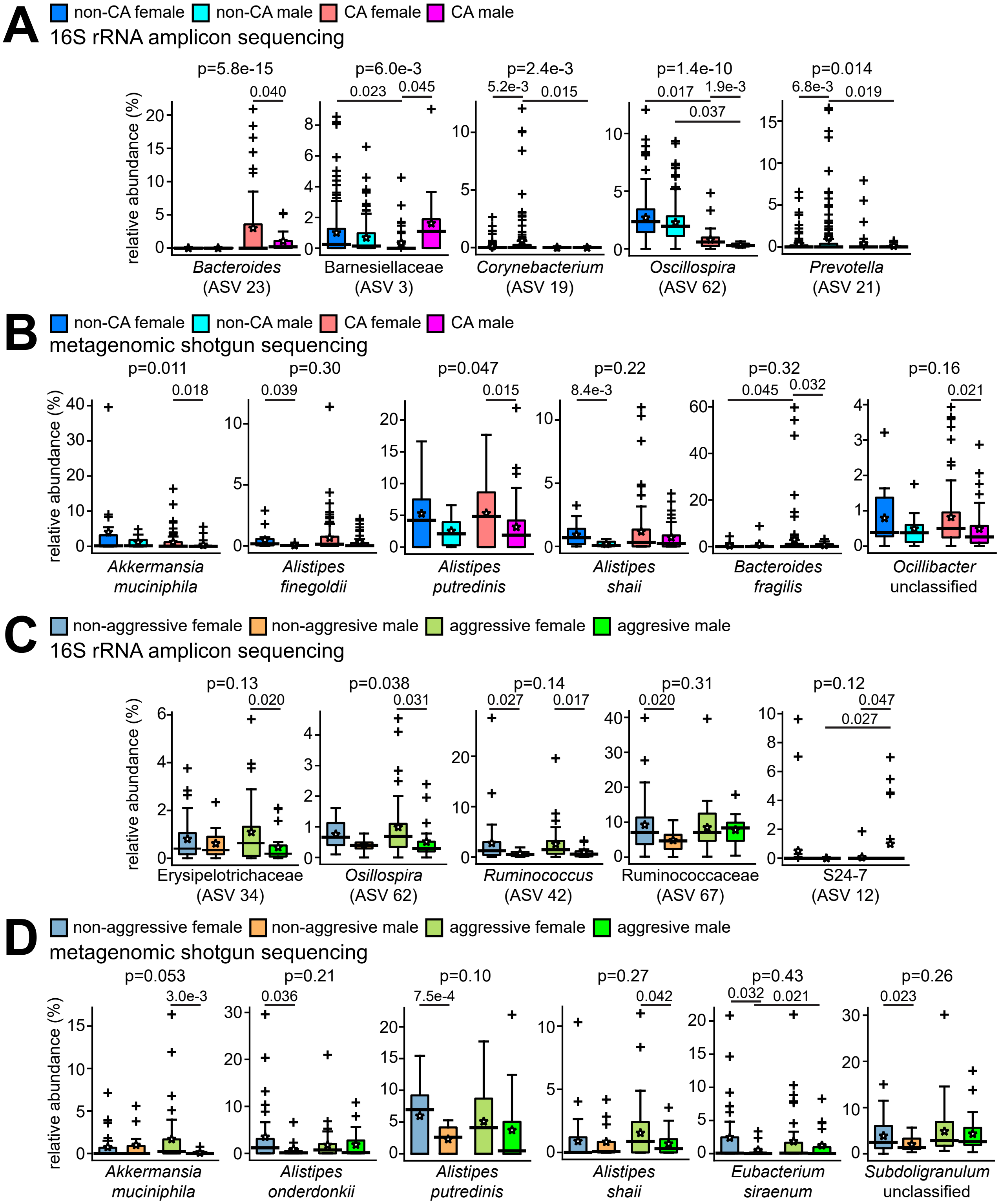
A
non-CA female
non-CA male
CA female
CA male
16S rRNA amplicon sequencing
relative abundance (%)
p=5.8e-15
0.040
Bacteroides (ASV 23)
p=6.0e-3
0.023
0.045
Barnesiellaceae (ASV 3)
p=2.4e-3
5.2e-3
0.015
Corynebacterium (ASV 19)
p=1.4e-10
0.017
1.9e-3
0.037
Oscillospira (ASV 62)
p=0.014
6.8e-3
0.019
Prevotella (ASV 21)
B
metagenomic shotgun sequencing
p=0.011
0.018
Akkermansia muciniphila
p=0.30
0.039
Alistipes finegoldii
p=0.047
0.015
Alistipes putredinis
p=0.22
8.4e-3
Alistipes shaii
p=0.32
0.045
0.032
Bacteroides fragilis
p=0.16
0.021
Ocillibacter unclassified
C
non-aggressive female
non-aggresive male
aggressive female
aggresive male
16S rRNA amplicon sequencing
p=0.13
0.020
Erysipelotrichaceae (ASV 34)
p=0.038
0.031
Osillospira (ASV 62)
p=0.14
0.027
0.017
Ruminococcus (ASV 42)
p=0.31
0.020
Ruminococcaceae (ASV 67)
p=0.12
0.047
0.027
S24-7 (ASV 12)
D
metagenomic shotgun sequencing
p=0.053
3.0e-3
Akkermansia muciniphila
p=0.21
0.036
Alistipes onderdonkii
p=0.10
7.5e-4
Alistipes putredinis
p=0.27
0.042
Alistipes shaii
p=0.43
0.032
0.021
Eubacterium siraenum
p=0.26
0.023
Subdoligranulum unclassified

Supplementary Figure 5. Gender differences of microbiome in CA patients.

A. Multi-group, multi-variate analysis of female and male fecal microbiome based on 16S rRNA gene amplicon sequencing data according to presence or absence of CA (n=128 for non-CA female, n=122 for non-CA male, n=80 for CA female, n=35 for CA male, ANCOM analysis followed by two-sided Mann-Whitney U test with Benjamini-Hochberg FDR correction). **B.** Multi-group, multi-variate analysis of female and male microbiome based on metagenomic shotgun sequencing data according to presence or absence of CA (n=17 for non-CA female, n=10 for non-CA male, n=86 for CA female, n=36 for CA male, ANCOM analysis followed by two-sided Mann-Whitney U test with Benjamini-Hochberg FDR correction). **C.** Multi-group, multi-variate analysis of female and male fecal microbiome based on 16S rRNA gene amplicon sequencing data according to presence or absence of aggressive CA (n=33 for non-aggressive female, n=10 for non-aggressive male, n=37 for aggressive female, n=21 for aggressive male, ANCOM analysis followed by two-sided Mann-Whitney U test with Benjamini-Hochberg FDR correction). **D.** Multi-group, multi-variate analysis of female and male fecal microbiome based on metagenomic shotgun sequencing data according to presence or absence of aggressive CA (n=34 for non-aggressive female, n=11 for non-aggressive male, n=41 for aggressive female, n=21 for aggressive male, ANCOM analysis followed by two-sided Mann-Whitney U test with Benjamini-Hochberg FDR correction). For all panels, p values for multi-group analyses, and significant p values in post hoc tests are shown. In box plots, bounds of boxes show IQR, top and bottom whiskers demonstrate maxinum and mininum, lines in the middle of the box indicate median, and stars show mean of the data. + signs indicate outliers.

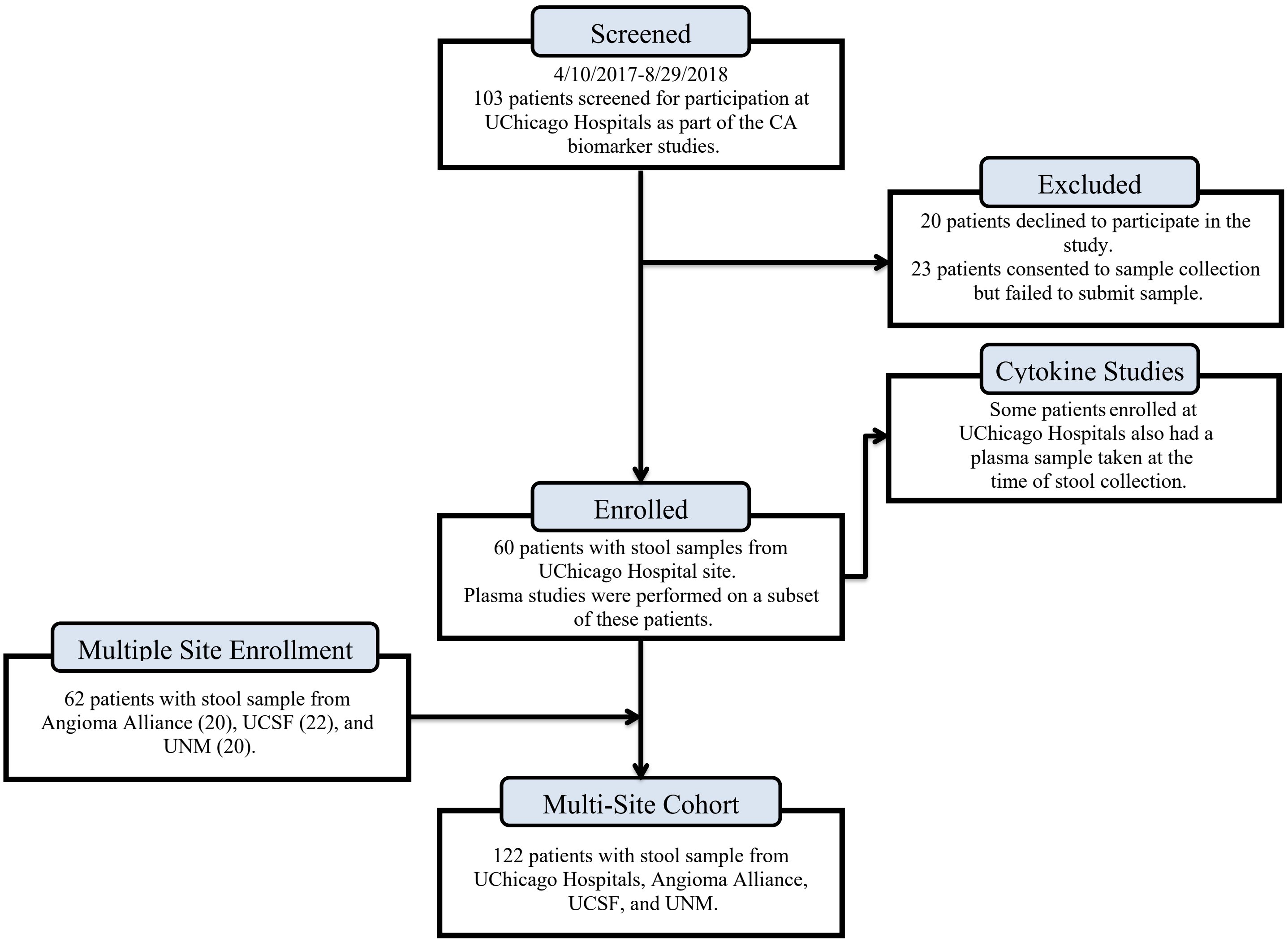
Screened
4/10/2017-8/29/2018
103 patients screened for participation at UChicago Hospitals as part of the CA biomarker studies.
Excluded
20 patients declined to participate in the study.
23 patients consented to sample collection but failed to submit sample.
Cytokine Studies
Some patients enrolled at UChicago Hospitals also had a plasma sample taken at the time of stool collection.
Enrolled
60 patients with stool samples from UChicago Hospital site.
Plasma studies were performed on a subset of these patients.
Multiple Site Enrollment
62 patients with stool sample from Angioma Alliance (20), UCSF (22), and UNM (20).
Multi-Site Cohort
122 patients with stool sample from UChicago Hospitals, Angioma Alliance, UCSF, and UNM.

Supplementary Figure 6. Patient enrollment and analysis CONSORT diagram.

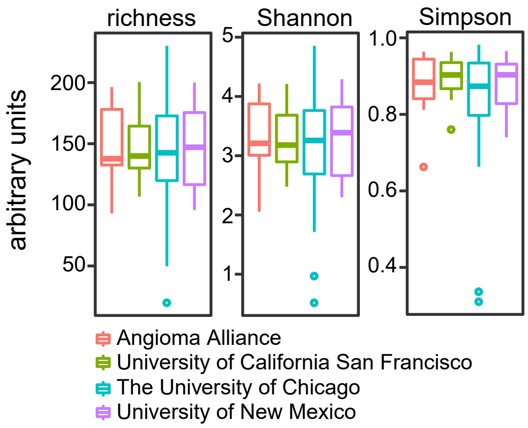
richness
Shannon
Simpson
arbitrary units
200
150
100
50
5
4
3
2
1
1.0
0.8
0.6
0.4
Angioma Alliance
University of California San Francisco
The University of Chicago
University of New Mexico

Supplementary Figure 7. Similar microbiome in samples collected at four sites.

α diversity analyses of fecal samples of CA patients from four different collection centers (n=19 for Angioma Alliance, n=21 for University of California San Francisco, n=55 for the University of Chicago, n=20 for University of New Mexico, Kruskal-Wallis one way analysis of variance test). In box plots, bounds of boxes show IQR, top and bottom whiskers demonstrate maxinum and mininum, lines in the middle of the box indicate median, and stars show mean of the data. + signs indicate outliers.

Supplementary Tables

	IFNg	IL-6	IL-10	IL-1b	TNF	CRP	TLR4	sCD14	LPB	VEGF	endoglin	THBS1	TM	
**s__Bacteroides_cellulosilyticus**	0.6986	-0.0035	0.07315	-0.1064	-0.055	-0.0061	-0.1405	0.1842	0.04533	0.35341	0.13664	-0.2346	0.0329	correlation coefficient
s__Bacteroides_cellulosilyticus	<.0001	0.9851	0.6958	0.5688	0.7687	0.9695	0.3572	0.2152	0.7729	0.0511	0.3598	0.1623	0.8467	p value
	31	31	31	31	31	42	45	47	43	31	47	37	37	n
**s__Bacteroides_dorei**	-0.112	0.05671	0.07457	-0.1322	0.07319	-0.0145	-0.0295	0.23014	-0.2042	0.19088	0.1269	0.01972	-0.0127	
s__Bacteroides_dorei	0.5478	0.7619	0.6901	0.4783	0.6956	0.9276	0.8474	0.1197	0.189	0.3037	0.3953	0.9078	0.9405	
	31	31	31	31	31	42	45	47	43	31	47	37	37	
**s__Bacteroides_eggerthii**	-0.068	-0.071	0.00454	-0.0512	-0.2058	0.03835	-0.0471	-0.1033	-0.0949	0.02753	0.17349	-0.0394	0.0896	
s__Bacteroides_eggerthii	0.716	0.7045	0.9806	0.7845	0.2666	0.8095	0.7589	0.4895	0.5451	0.8831	0.2435	0.8168	0.5979	
	31	31	31	31	31	42	45	47	43	31	47	37	37	
**s__Bifidobacterium_adolescentis**	-0.074	0.32674	-0.0852	0.47557	-0.1288	0.00429	0.07809	0.20024	0.06711	-0.2922	0.01891	-0.0107	0.28404	
s__Bifidobacterium_adolescentis	0.6929	0.0728	0.6487	0.0069	0.49	0.9785	0.6101	0.1772	0.669	0.1107	0.8996	0.9498	0.0884	
	31	31	31	31	31	42	45	47	43	31	47	37	37	
**s__Dorea_unclassified**	0.0307	0.0581	-0.0337	0.00883	-0.1563	-0.0508	-0.0216	-0.0236	-0.0796	-0.0602	0.09471	0.15548	-0.2234	
s__Dorea_unclassified	0.8697	0.7562	0.8573	0.9624	0.4013	0.7494	0.8879	0.8751	0.612	0.7479	0.5266	0.3581	0.1838	
	31	31	31	31	31	42	45	47	43	31	47	37	37	
**s__Enterobacter_cloacae**	.	.	.	.	.	.	-0.0522	-0.1682	.	.	0.07469	0.02084	0.07366	
s__Enterobacter_cloacae	.	.	.	.	.	.	0.7333	0.2585	.	.	0.6178	0.9026	0.6648	
	31	31	31	31	31	42	45	47	43	31	47	37	37	
**s__Escherichia_coli**	-0.082	-0.1183	-0.0836	-0.1695	0.16161	-0.146	0.17683	-0.2742	0.28445	-0.2232	-0.063	-0.0031	-0.1251	
s__Escherichia_coli	0.6601	0.5262	0.655	0.3621	0.3851	0.3563	0.2452	0.0622	0.0645	0.2274	0.6739	0.9854	0.4605	
	31	31	31	31	31	42	45	47	43	31	47	37	37	
**s__Faecalibacterium_prausnitzii**	-0.086	0.12071	0.23674	0.1965	0.04348	-0.136	-0.0205	-0.0589	-0.3316	-0.0849	-0.3119	0.29911	-0.2754	
s__Faecalibacterium_prausnitzii	0.6447	0.5177	0.1997	0.2894	0.8163	0.3904	0.8936	0.6943	0.0298	0.6499	0.0329	0.0721	0.099	
	31	31	31	31	31	42	45	47	43	31	47	37	37	
**s__Lactobacillus_rhamnosus**	-0.044	-0.0758	-0.0625	-0.0563	0.11243	-0.0876	-0.0505	0.1937	-0.1621	-0.145	0.10912	-0.0084	-0.0281	
s__Lactobacillus_rhamnosus	0.8138	0.6851	0.7382	0.7635	0.547	0.581	0.742	0.192	0.2991	0.4364	0.4653	0.9607	0.8687	
	31	31	31	31	31	42	45	47	43	31	47	37	37	
**s__Odoribacter_laneus**	-0.056	-0.0523	-0.0682	-0.1215	0.12705	-0.1177	-0.0354	-0.072	-0.0557	0.06154	-0.1527	-0.1924	0.02375	
s__Odoribacter_laneus	0.7647	0.7798	0.7154	0.5149	0.4958	0.458	0.8177	0.6308	0.7227	0.7422	0.3056	0.2539	0.889	
	31	31	31	31	31	42	45	47	43	31	47	37	37	
**s__Odoribacter_splanchnicus**	0.0349	-0.1113	0.09291	-0.05	-0.0927	-0.093	-0.0535	-0.1591	-0.2022	-0.28	-0.1778	0.00343	-0.1674	
s__Odoribacter_splanchnicus	0.852	0.551	0.6191	0.7895	0.6201	0.5581	0.7272	0.2854	0.1934	0.1271	0.2318	0.9839	0.322	
	31	31	31	31	31	42	45	47	43	31	47	37	37	
**s__Oscillibacter_unclassified**	-0.013	0.09022	-0.0377	0.16693	0.50762	-0.239	0.54661	0.05435	0.38985	0.00834	-0.1364	-0.0937	-0.0072	
s__Oscillibacter_unclassified	0.9426	0.6293	0.8404	0.3694	0.0036	0.1275	0.0001	0.7167	0.0098	0.9645	0.3607	0.5813	0.9663	
	31	31	31	31	31	42	45	47	43	31	47	37	37	

	IFNg	IL-6	IL-10	IL-1b	TNF	CRP	TLR4	sCD14	LPB	VEGF	endoglin	THBS1	TM	
**non-CA vs CA species**	-0.074	0.13259	0.19211	0.23752	-0.0151	-0.117	0.02105	0.04327	-0.2458	-0.196	-0.2569	0.20045	-0.0501	correlation coefficient
	0.6932	0.4771	0.3005	0.1982	0.9357	0.4606	0.8908	0.7727	0.1122	0.2908	0.0813	0.2342	0.7685	p value
	31	31	31	31	31	42	45	47	43	31	47	37	37	n
**non-aggressive vs aggressive spec**	0.0196	0.1394	-0.0729	0.12369	0.07989	-0.0699	0.10694	0.11712	0.12256	-0.2081	-0.0344	0.07294	0.04405	
	0.9167	0.4545	0.6966	0.5074	0.6692	0.6601	0.4844	0.433	0.4336	0.2613	0.8186	0.6679	0.7957	
	31	31	31	31	31	42	45	47	43	31	47	37	37	
**non-CASH vs CASH species**	-0.178	0.15617	0.1529	0.27414	0.33692	-0.2388	0.30804	-0.0458	-0.0459	-0.0973	-0.3675	0.20021	-0.2262	
	0.3391	0.4015	0.4116	0.1356	0.0638	0.1277	0.0395	0.7598	0.7699	0.6026	0.011	0.2348	0.1783	
	31	31	31	31	31	42	45	47	43	31	47	37	37	

Supplementary Table 1. Correlation between bacterial species and plasma biomarkers.

**Patient Characteristics**							
Group (n=122)	Total (122)	Sporadic/ Solitary (29)	Familial/Multifocal Combined total (93)	Familial/Multifocal Subgroups			
				CCM 1 (45)	*CCM2 (13)*	*CCM 3 (9)*	Unknown Genotype (26)
Age Years- Mean (SD)	41.68 (18.71)	45.51 (15.56)	40.49 (19.51)	39.48 (20.23)	38.23 (23.71)	37.64 (16.27)	44.84 (16.96)
Female	86	20	66	38	6	7	16
Aggressive Features$^{\psi}$	62	7	55	30	4	5	16
• 1st Hemorrhage ≤18 years old	23	3	20	11	2	3	4
• ≥5 T_2-weighted lesions	NA*	NA*	28	16	1	3	8
• ≥25 lesions on SWI	NA*	NA*	28	17	2	3	6
• ≥2 adjudicated symptomatic hemorrhages	16	4	12	4	1	0	7
T_2 Lesion Count- mean (SD)	NA*	1*	10.36 (42.15)	4.61 (5.35)	3.75 (3.57)	15.00 (6.56)	4.26 (4.05)
SWI Lesion Count- mean (SD)	NA*	1*	49.20 (74.67)	66.00 (91.10)	14.00 (16.9)	54.00 (40.78)	35.52 (56.02)
CASH (%)	13 (10.7)	5 (17.2)	8 (8.6)	3 (6.67)	0	1 (11.1)	4 (15.4)
**Microbiome Survey Response****							
Self-reported inflammatory bowel disease (IBD)	11.48% (14/122)	17.24 % (5/29)	9.68 % (9/93)	11.12 % (5/45)	7.69 % (1/13)	11.11 % (1/9)	7.69% (2/26)
Self-reported antibiotics usage within prior 6 months	27.50% (22/80)	24.13 % (7/29)	29.41% (15/51)	13.51 % (5/37)	9.09 % (1/11)	33.34 % (1/3)	38.10 % (8/21)

ψ Aggressive features are defined as patients have at least one of the four components (Mikati et al.).
* Lesion count in the sporadic from of the disease is always one except in rare cases.
** Microbiome Survey completed at the time of stool collection, denominator represents those that completed the survey.
SWI- susceptibility weighted images
SD- standard deviation
CASH- CA with symptomatic hemorrhage

Supplementary Table 2. CA Patient Characteristics

**Screened Patient Characteristics-Not enrolled**						
Group (n=43 total)	Sporadic/ Solitary (31)	Familial/Multifocal Combined total (12)	Familial/Multifocal Subgroups			
			CCM 1 (3)	*CCM2 (4)*	*CCM 3 (3)*	Unknown Genotype (2)
Age Years- Mean (SD)	45.4 (11.8)	34.7 (13.6)	39.3 (19.1)	38.5 (14.4)	29.0 (11.5)	28.5 (10.6)
Female (%)	21(67.7)	9 (75)	2 (66.7)	4 (100)	3 (100)	0
Aggressive Featuresψ	7	3	1	0	2	0
• 1st Hemorrhage ≤18 years old	0	1	0	0	1	0
• ≥5 T_2-weighted lesions	NA*	3	1	0	2	0
• ≥25 lesions on SWI	NA*	2	1	0	1	0
• ≥2 adjudicated symptomatic hemorrhages	4	1	1	0	0	0
T_2 Lesion Count- mean (SD)	1*	4.3 (5.5)	6.3 (5.9)	1.0 (0.82)	8.7 (8.1)	1.5 (0.7)
SWI Lesion Count- mean (SD)	1*	17.1 (29.9)	42 (55.7)	1.0 (0.82)	20.3 (15.4)	7.0 (1.4)

ψ Aggressive features are defined as patients have at least one of the four components (Mikati et al.).
* Lesion count in the sporadic from of the disease is always one except in rare cases not observed in this series
SD- standard deviation
SWI- susceptibility weighted images
ND- no data

Supplementary Table 3. CA Patient Characteristics of Screened Cases Not Enrolled

							Familial/Multifocal		
**Site**	n=	Average Age (SD)	Female	Sporadic /Solitary	Aggressive	CASH	Familial/Multifocal (Genotype CCM1/2/3/unknown)	Average T2 Count (SD)	Average SWI Count (SD)
University of Chicago	60	46.2 (15.5)	41	28	24	9	32 (11/7/3/11)	3.8 (5.2)	19.1 (33.9)
Angioma Alliance	20	28.9 (16.00)	13	0	11	0	20 (7/4/6/3)	63.6 (130.7)	ND
UCSF	22	41.8 (21.4)	15	1	16	2	21 (7/1/0/7)	3.1 (3.4)	61.2 (95.2)
UNM	20	38.9 (21.6)	17	0	11	2	20 (20/0/0/0)	3.9 (3.8)	68.06 (96.1)

SD- standard deviation
ND- no data

Supplementary Table 4. Demographics Based on Site of Collection
