# Supplementary material for: Permissive microbiome characterizes human subjects with a neurovascular disease cavernous angioma
Source: Nat Commun. 2020 May 27;11:2659. doi: 10.1038/s41467-020-16436-w (PMC7253448; doi:10.1038/s41467-020-16436-w)
Supplement: Supplementary file 2 — Reporting Summary [file 41467_2020_16436_MOESM2_ESM.pdf]

nature research

Corresponding author(s): Le Shen, Issam Awad

Last updated by author(s): Apr 5, 2020

Reporting Summary

Nature Research wishes to improve the reproducibility of the work that we publish. This form provides structure for consistency and transparency in reporting. For further information on Nature Research policies, see Authors & Referees and the Editorial Policy Checklist .

Statistics

For all statistical analyses, confirm that the following items are present in the figure legend, table legend, main text, or Methods section.

n/a	Confirmed	
☐	☒	The exact sample size (*n*) for each experimental group/condition, given as a discrete number and unit of measurement
☐	☒	A statement on whether measurements were taken from distinct samples or whether the same sample was measured repeatedly
☐	☒	The statistical test(s) used AND whether they are one- or two-sided<br>*Only common tests should be described solely by name; describe more complex techniques in the Methods section.*
☐	☒	A description of all covariates tested
☐	☒	A description of any assumptions or corrections, such as tests of normality and adjustment for multiple comparisons
☐	☒	A full description of the statistical parameters including central tendency (e.g. means) or other basic estimates (e.g. regression coefficient) AND variation (e.g. standard deviation) or associated estimates of uncertainty (e.g. confidence intervals)
☐	☒	For null hypothesis testing, the test statistic (e.g. *F*, *t*, *r*) with confidence intervals, effect sizes, degrees of freedom and *P* value noted<br>*Give P values as exact values whenever suitable.*
☒	☐	For Bayesian analysis, information on the choice of priors and Markov chain Monte Carlo settings
☒	☐	For hierarchical and complex designs, identification of the appropriate level for tests and full reporting of outcomes
☐	☒	Estimates of effect sizes (e.g. Cohen's *d*, Pearson's *r*), indicating how they were calculated

Our web collection on statistics for biologists contains articles on many of the points above.

Software and code

Policy information about availability of computer code

Data collection	RedCap and Excel
Data analysis	16S data analyses-<br>-16S rRNA gene sequencing data from enrolled CA patients and American Gut Project non-CA were quality-filtered and demultiplexed using QIIME 2_v2018.11.<br>-Demultiplexed sequences were then selected for ESV picking by using DeBlur trimmed to 125 nucleotides. ESVs present in less than 10 samples were removed by using Phyloseq_v1.26.1.<br>-The final BIOM file comprising of unique 60,141 ESVs with average 32,190 reads per sample was then used for further analyses.<br>-Richness, Shannon and Simpson indices were used to estimate alpha diversity and the variation between groups (beta diversity) was statistically tested by using permutational multivariate analysis of variance (PERMANOVA) test in package phyloseq_v1.26.1 in R_v3.5.1.<br>-ANCOM_v2 was used to identify differentially abundant bacterial ESVs between the groups at P-value cut-off of 0.05 with Benjamini-Hochberg FDR correction.<br>-The confounding variable i.e. age, gender and collection site were adjusted for in the ANCOM2 analyses.<br>-ANCOM2 results were plotted using box-plots in R_v3.5.1.<br>-Spearman rank correlation and generalized linear models (GLMs) were used to establish association between the microbiome and other continuous variables in the metadata using microbiomeSeq()_0.1 and glm()_v1.2.1 packages in R_v3.5.1.<br><br>Metagenomic shotgun sequencing analysis-<br>-For metagenomic shotgun sequencing analysis, 2.2 billion paired-end metagenome reads were quality trimmed (for adapters, primers and oligonucleotides) with Nesoni2.<br>-To assess taxonomic diversity, trimmed data were analyzed using MetaPhlAn2 to profile the composition of microbial communities (bacteria, archaea, eukaryotes and viruses) at species level. A database of ~1M unique clade-specific marker genes identified from ~17,000 reference genomes were used in MetaPhlAn2, and BowTie2 was used for reference-based alignment of the reads.

-Co-occurrence networks were generated by calculating Spearman's correlations between abundance of species using Hmisc_v4.2.0 in R_v3.5.1. -Significant connections (Benjamini-Hochberg FDR corrected p-value < 0.05) were exported as GML format network files using igraph_v1.2.1 in R_v3.5.1.
-ANCOM2 was used to identify differentially abundant bacterial species between the groups at p value cut-off of 0.05 with Benjamini-Hochberg FDR correction. The confounding variable i.e. age, gender and collection site were adjusted for in the ANCOM2 analyses.
-Functional profiling was performed using HUMAnN2 which identifies the species profile from metagenomic shotgun sequencing data and aligns reads to their pangenomes, performs translated search on unclassified reads, and quantifies gene families and pathways.
-Differential analyses and plotting was performed using ANCOM2. Age, gender and sites were adjusted for in all the analyses.
- Random forest algorithm was used to extract the most important features in the microbiome species information by using the Boruta pipeline.
-Spearman rank correlation and generalized linear models (GLMs) were used to establish association between the microbiome and other continuous variables in the metadata using microbiomeSeq()_v0.1 and glm()_v1.2.1 packages in R_3.5.1.
-For control population, a control cohort stool samples banked at the University of Pennsylvania Microbiome Center were processed, sequenced, and analyzed along with CA cohort samples and sequencing results were corrected for age and gender during analysis.

Correlation between microbiome and plasma biomarkers and ROC curve were performed in SAS9.4

Microbiome random forest analysis were performed by Baruta package v6.0.0 in R_3.5.1

For manuscripts utilizing custom algorithms or software that are central to the research but not yet described in published literature, software must be made available to editors/reviewers. We strongly encourage code deposition in a community repository (e.g. GitHub). See the Nature Research guidelines for submitting code & software for further information.

Data

Policy information about availability of data

All manuscripts must include a data availability statement. This statement should provide the following information, where applicable:

- Accession codes, unique identifiers, or web links for publicly available datasets
- A list of figures that have associated raw data
- A description of any restrictions on data availability

The datasets generated are deposited in European Nucleotide Archive.

Field-specific reporting

Please select the one below that is the best fit for your research. If you are not sure, read the appropriate sections before making your selection.

☒ Life sciences ☐ Behavioural & social sciences ☐ Ecological, evolutionary & environmental sciences

For a reference copy of the document with all sections, see nature.com/documents/nr-reporting-summary-flat.pdf

Life sciences study design

All studies must disclose on these points even when the disclosure is negative.

Sample size	No sample size calculation was performed. Sample size was estimated based on previous human microbiome studies, and the goal was to ensure the smallest sub-category of patients have n ~10 patients.
Data exclusions	The quality filtering was done at the time of raw data processing as well as biome processing for statistical analyses based on the criterion mentioned in the methods section of the manuscript. Samples failed these thresholds were excluded in the analyses. For plasma biomarker studies, values away from 2SD were excluded.
Replication	For microbiome analyses, in order to maintain reproducibility, we ran duplicate samples across two runs of the microbiome sequencing. All replication are successful. Additionally, to supplement results from 16S rRNA based sequencing, we performed in-depth shotgun metagenomics. Furthermore, we also performed additional analyses to supplement one analyses with another. For instance, multi-variate differential abundance analyses was validated using machine learning based random forest analyses and for each model accuracy as well as sensitivity was calculated to support the credibility of the biomarkers identfied.
Randomization	No randomization was performed, because it was a cross sectional cohort study. Patients were sequentially enrolled irrespective to their genotype and phenotype.
Blinding	Stool samples were collected, processed, and sequenced in a blinded fashion. Bioinformatic analysis (ESVs and species calling) were also performed blindly. Investigators were blinded to group allocation during data analysis.

Reporting for specific materials, systems and methods

We require information from authors about some types of materials, experimental systems and methods used in many studies. Here, indicate whether each material, system or method listed is relevant to your study. If you are not sure if a list item applies to your research, read the appropriate section before selecting a response.

Materials & experimental systems

n/a	Involved in the study
☐	☒ Antibodies
☒	☐ Eukaryotic cell lines
☒	☐ Palaeontology
☒	☐ Animals and other organisms
☐	☒ Human research participants
☐	☒ Clinical data

Methods

n/a	Involved in the study
☒	☐ ChIP-seq
☒	☐ Flow cytometry
☐	☒ MRI-based neuroimaging

Antibodies

Antibodies used: anti-IFN-γ, anti-IL-10, anti-IL-1β, anti-IL-6, anti-TNF-α, anti-VEGF-A (all MSD), anti-TLR4 (Raybiotech), anti-LPB (R&D Systems), anti-CRP (R&D systems), anti-sCD14 (R&D Systems), anti-endoglin (R&D systems), anti-thrombomodulin (R&D systems), anti-thrombospondin-1 (R&D systems).

Validation: Antibodies were validated by the manufacturers and were provided as part of ELISA kits.

Human research participants

Policy information about studies involving human research participants

Population characteristics: full population characteristics are provided in the manuscript in Online material and methods, and in supplementary Tables 1 to 3.

Recruitment: Patients were recruited during regular clinical visit or through phone calls. Inclusion criteria were a clinical index diagnosis of CA where at least one brain lesion had not been resected. Supplementary Table 3 shows patients not enrolled for the study have similar characteristics relative to patients participated in the study.

Ethics oversight: The University of Chicago, University of New Mexico, University of California San Francisco, Angioma Alliance

Note that full information on the approval of the study protocol must also be provided in the manuscript.

Clinical data

Policy information about clinical studies

All manuscripts should comply with the ICMJEguidelines for publication of clinical research and a completedCONSORT checklist must be included with all submissions.

Clinical trial registration: Clinical trial registration was not performed because it is not a clincal trial, no intervention was performed.

Study protocol: Clinical trial study protocol is not included because it is not a clincal trial.

Data collection: Patients were recruited from April 10, 2017 to August 29, 2018, Stool samples were collected at patients home. Clinical information and blood samples for these patients were collected during patients' clinical visits at participating sites.

Outcomes: Primary and secondary outcomes were not listed because they were not set ahead of time, as it was not a clinical trial.

Magnetic resonance imaging

Experimental design

Design type: Observational cross sectional cohort study.

Design specifications: MR imaging was performed as part of standard CA clinical services. No addition

Behavioral performance measures: none.

Acquisition

Imaging type(s)	Structural imaging
Field strength	3 T
Sequence & imaging parameters	T2 weighted and SWI (susceptibility weighted imaging)
Area of acquisition	Whole brain

Diffusion MRI ☐ Used ☒ Not used

Preprocessing

Preprocessing software	None
Normalization	None
Normalization template	None
Noise and artifact removal	None
Volume censoring	None

Statistical modeling & inference

Model type and settings	None
Effect(s) tested	None

Specify type of analysis: ☐ Whole brain ☐ ROI-based ☐ Both

Statistic type for inference (See Eklund et al. 2016)	None
Correction	None

Models & analysis

n/a	Involved in the study
☒	☐ Functional and/or effective connectivity
☐	☒ Graph analysis
☒	☐ Multivariate modeling or predictive analysis

Graph analysis	Lesion size and number were measured manually by clinician off DICOM images
